# Overlapping RAD18-and DNA-binding interfaces in DNA polymerase η contribute to UV-induced DNA damage tolerance

**DOI:** 10.1016/j.jbc.2026.113555

**Published:** 2026-09-15

**Authors:** Rika Kusumoto-Matsuo, Yuji Masuda, Rie Kanao, Shigenori Iwai, Chikahide Masutani

**Affiliations:** 1 Department of Genome Dynamics, Research Institute of Environmental Medicine, Nagoya University, Nagoya, Japan; 2 Advanced Cancer Translational Research Institute, Showa Medical University, Tokyo, Japan; 3 Department of Molecular Pharmaco-Biology, Nagoya University Graduate School of Medicine, Nagoya, Japan; 4 Research Institute for Radiation Biology and Medicine, Hiroshima University, Hiroshima, Japan; 5 Division of Chemistry, Graduate School of Engineering Science, Osaka University, Osaka, Japan; 6 Biosignal Research Center, Kobe University, Kobe, Hyogo, Japan; 7 Frontiers of Innovative Research in Science and Technology, Konan University, Kobe, Hyogo, Japan

**Keywords:** DNA damage response, DNA replication, DNA polymerase η, RAD18, ubiquitination

## Abstract

DNA polymerase η (Polη) bypasses UV-induced pyrimidine dimers and thereby confers tolerance to UV irradiation. Although the C-terminus of Polη has been reported to interact with ubiquitinated PCNA and RAD18, how Polη engages RAD18 is not fully understood. Here, we show that Polη and RAD18 interact through two distinct modes in human cells: a ubiquitinated-PCNA-dependent mode that requires the Polη C-terminus, and an unexpected PCNA-independent mode mediated by its N-terminal region. We focused our subsequent analyses on this newly identified PCNA-independent mode. Using purified recombinant proteins, we demonstrate direct binding of the N-terminal region of human Polη (PolηΔC) to RAD18 *in vitro*. Although PolηΔC and RAD18 each bound primer-template DNA, we were unable to detect a ternary PolηΔC-RAD18-DNA complex, and DNA competitively inhibited RAD18 binding to both PolηΔC and full-length Polη. Mutational analyses revealed that the DNA-binding and RAD18-binding domains within Polη overlap. A separation-of-function mutant, PolηΔC(K317A), which retains near-normal DNA-binding and polymerase activities but exhibits reduced RAD18 binding *in vitro,* displayed a diminished ability to rescue the UV sensitivity of Polη-deficient cells. Notably, the detrimental impact of the K317A persisted in a PCNA-binding-defective background but was attenuated in RAD18-KO cells. These findings demonstrate that RAD18 binding to the N-terminal domain of Polη contributes to efficient bypass of pyrimidine dimers independently of the Polη-PCNA interaction and provide mechanistic insights into how Polη-RAD18 complexes assemble and dissociate during translesion DNA synthesis.

Replicative DNA polymerases stall at sites of DNA damage, such as those caused by UV irradiation, reactive oxygen species, and chemical reagents. To cope with such replication-blocking lesions, cells have evolved mechanisms that confer tolerance to DNA damage. One major mechanism is translesion DNA synthesis (TLS), which is carried out by specialized DNA polymerases, including DNA polymerase η (Polη), Polι, Polκ, REV1, and Polζ (1, 2). However, recent studies have shown that additional DNA damage tolerance pathways, including polymerase-mediated mechanisms distinct from canonical TLS, as well as non-TLS pathways, also contribute to lesion bypass (3, 4, 5). Among TLS polymerases, Polη accurately and efficiently bypasses cyclobutane pyrimidine dimers (CPD), a major UV-induced lesion that is poorly repaired by nucleotide excision repair (6, 7, 8). In humans, loss of Polη function results in xeroderma pigmentosum variant (XP-V), an autosomal recessive disorder characterized by extreme sensitivity to sunlight and predisposition to UV-induced skin cancer (9, 10, 11).

Polη incorporates adenines opposite thymine-thymine CPDs (TT-CPDs) with high efficiency; however, its fidelity on undamaged DNA is substantially lower than that of replicative DNA polymerases (12). Consequently, Polη activity is thought to be largely restricted to sites of DNA damage. The DNA-binding region of Polη resides within its N-terminal domain, and Polη binds most stably to primer-template DNA when an adenine is positioned at the 3′ end of the primer opposite the TT-CPD in the template. The PolηΔC-primer-template DNA complex becomes destabilized after two nucleotides are inserted beyond the TT-CPD (13). Thus, Polη appears to be optimized to extend DNA synthesis just enough to bypass the lesion, but does so with limited fidelity (14).

Like Polη, RAD18 belongs to the RAD6 epistatic group (15). RAD18, an E3 ubiquitin ligase, forms a complex with RAD6, an E2 ubiquitin-conjugating enzyme (16, 17, 18, 19); in human cells, this complex has been determined to adopt a (RAD18)_2_-RAD6 stoichiometry (20, 21). The complex monoubiquitinates proliferating cell nuclear antigen (PCNA) at K164 and, in the presence of additional factors, can also promote PCNA polyubiquitination in response to replication fork stalling induced by DNA-damaging agents (22, 23, 24, 25, 26, 27, 28). Monoubiquitination of PCNA by the RAD18-RAD6 complex plays a central role in TLS (23, 29, 30, 31) and is dynamically reversed by deubiquitinating enzymes to prevent excessive activation of error-prone TLS polymerases (32, 33).

Protein-protein interactions, particularly those involving Polη, monoubiquitinated PCNA, and RAD18, are crucial for the recruitment and accumulation of Polη around sites of stalled DNA replication (23, 29, 34). Polη contains three, noncanonical PCNA-interacting protein (PIP) boxes and an ubiquitin-binding zinc-finger (UBZ) domain (35). Although the PIP motifs have distinct roles in stimulating DNA synthesis and promoting PCNA monoubiquitination, they contribute redundantly to cell viability after UV irradiation (35). In addition to monoubiquitinated PCNA, Polη interacts with REV1 *via* its two mutually redundant REV1-interacting regions, enhancing its accumulation around sites of stalled replication (36, 37). These multiple interactions are thought to coordinate the bypass activities of various TLS polymerases with different cognate lesion specificities (38).

Among TLS polymerases, Polη uniquely interacts with RAD18. The interaction contributes to the activities of both proteins: Polη colocalizes with RAD18 upon UV irradiation (34, 39), and their association in the chromatin fraction is also enhanced following UV exposure (40). The C-terminal region of RAD18 mediates its interaction with Polη, and this interaction is further strengthened by RAD18 phosphorylation (29, 39, 41). Conversely, Polη promotes RAD18-mediated monoubiquitination of PCNA through its PIP motifs (35), leading to increased accumulation of ubiquitinated PCNA and Polη around sites of arrested DNA replication. Pull-down assays using cell lysates have indicated that the C-terminal region of Polη (amino acids 556–713) contains a RAD18 binding domain (29).

In this study, we sought to clarify how Polη and RAD18 cooperate during the early steps of DNA damage tolerance. Our analyses revealed that Polη and RAD18 interact through two distinct modes in cells: a ubiquitinated-PCNA-dependent mode that requires the C-terminal region of Polη, and an unexpected PCNA-independent mode mediated by its N-terminal region. We further found that the Polη-RAD18 complex does not assemble on DNA; instead, DNA binding by Polη interferes with RAD18 association due to overlapping DNA- and RAD18-binding regions within the Polη N-terminus. Finally, we show that disruption of this interaction by a point mutation compromises cellular resistance to UV irradiation. We discuss the biological significance of these two modes of Polη-RAD18 interaction.

## Results

### Ubiquitinated PCNA-dependent and -independent modes of Polη-RAD18 interaction in cells

Ectopic expression of Polη robustly induces RAD18-mediated monoubiquitination of PCNA even in the absence of UV irradiation in human cells (34, 35), and RAD18 interacts preferentially with Polη rather than with other TLS polymerases (42). These observations prompt us to assess the functional interplay among Polη, RAD18, and monoubiquitinated PCNA. We therefore first examined whether PCNA monoubiquitination influences the interaction between Polη and RAD18 using cells in which endogenous PCNA was depleted by siRNA and exogenous WT PCNA (PCNA[WT]) or a PCNA mutant in which lysine 164 was substituted with arginine (PCNA[KR]) was expressed (Figs. 1 and S1) (43). Ectopic expression of N-terminally FLAG-tagged full-length Polη (^FLAG^PolηFL) increased the level of monoubiquitinated PCNA in the chromatin fraction of PCNA[WT] cells (Fig. 1*B*). Under these conditions, RAD18 as well as monoubiquitinated PCNA were co-precipitated with ^FLAG^PolηFL in PCNA[WT] cells, indicating that Polη associates with both RAD18 and monoubiquitinated PCNA in the chromatin fraction. In contrast, expression of PCNA[KR] abolished PCNA monoubiquitination and markedly reduced the interaction between ^FLAG^PolηFL and RAD18 in the chromatin fraction (Figs. 1*B* and S1*B*). However, substantial amounts of RAD18 were co-precipitated with ^FLAG^PolηFL in the detergent-soluble fraction, where monoubiquitinated PCNA was scarcely detected, regardless of whether cells expressed PCNA[WT] or PCNA[KR] and irrespective of UV irradiation (Figs. 1*A* and S1*A*).

**Figure 1:**
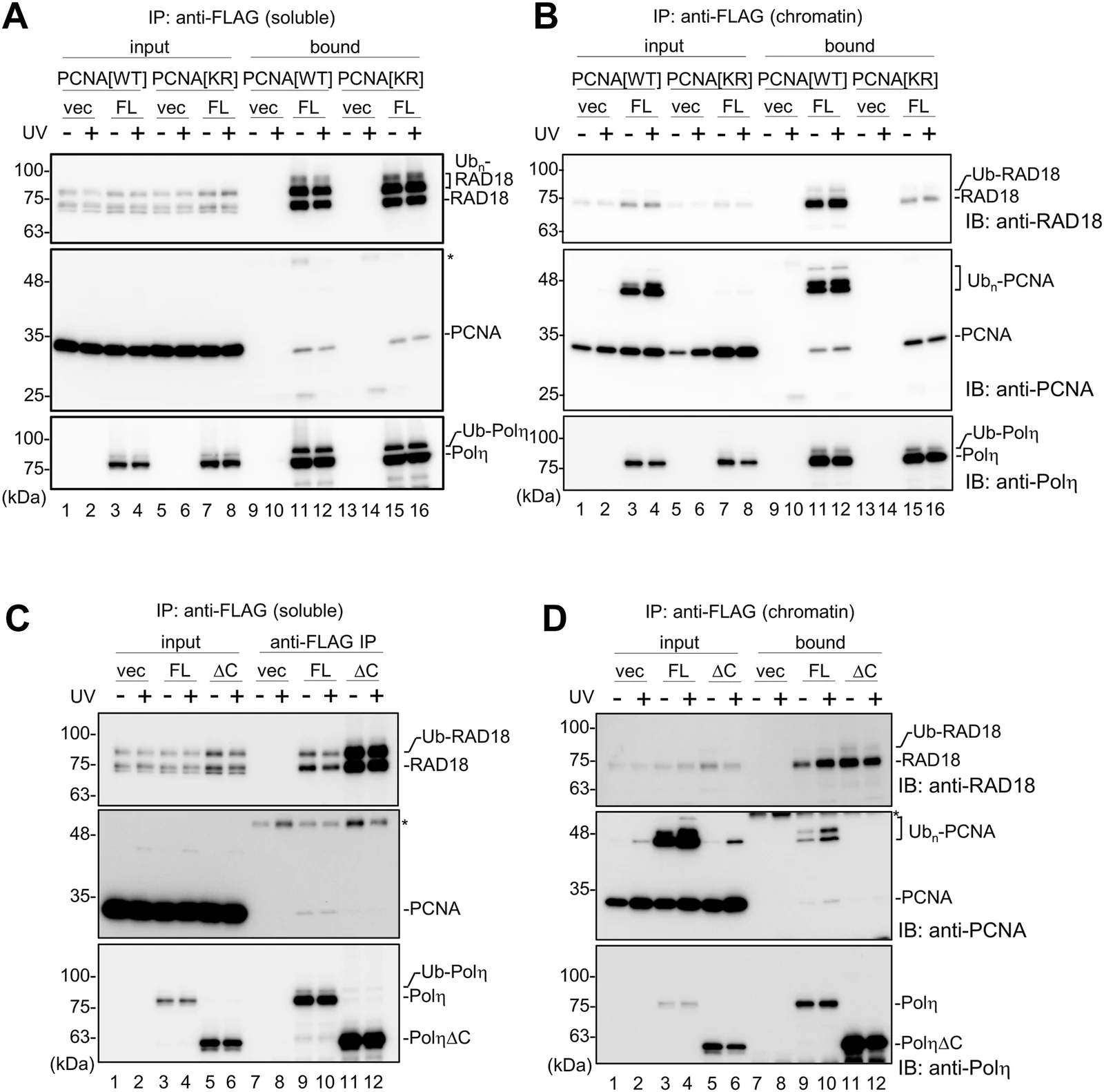
**RAD18-binding activity of Polη in cells.** Anti-FLAG immunoprecipitation. *A* and *B,* XP2SASV3 cells stably expressing exogenous PCNA[WT] or PCNA[KR] were co-transfected with siRNA targeting endogenous PCNA and either an empty vector or a vector to express ^FLAG^Polη. Cells were irradiated with 15 J/m^2^ UV-C or mock-treated and incubated for 3 h. ^FLAG^Polη in detergent-soluble *(A)* and chromatin fractions *(B)* were immunoprecipitated with anti-FLAG antibody. Immunoprecipitated complexes (lanes 9–16) and 5% of input (lanes 1–8) used in immunoprecipitation were analyzed by Western blotting with indicated antibodies. *C and D,* XP2SASV3 cells were transfected with an empty vector or a vector to express ^FLAG^PolηFL or ^FLAG^PolηΔC, and subjected as in *(A and B, respectively)*. Immunoprecipitated complexes (lanes 7–12) and 5% of input (lanes 1–6) used in immunoprecipitation were analyzed by Western blotting with indicated antibodies. *A, C and D*, the positions of the immunoglobulin G heavy chain derived from the immunoprecipitating antibody are indicated as *asterisks*.

We next examined the interaction using a C-terminally truncated form of Polη (amino acids 1–511; Fig. 2*A*), hereafter referred to as PolηΔC. PolηΔC lacks both PIP2 motif and the UBZ domain, which are largely responsible for stimulating RAD18-mediated PCNA monoubiquitination (35) and for ubiquitin binding (44), respectively. This truncated form also lacks the previously reported interaction region with RAD18 (29). These assays were performed in cells expressing endogenous PCNA. As we reported previously (35), expression of the ^FLAG^PolηΔC resulted in much lower levels of PCNA monoubiquitination than ^FLAG^PolηFL (Fig. 1*D*). Surprisingly, however, RAD18 was prominently co-precipitated with ^FLAG^PolηΔC in both the soluble and chromatin fraction (noting that ^FLAG^PolηΔC was expressed at high levels, which precludes quantitative comparison) (Figs. 1*C*, D, S1*C*, and *D*).

**Figure 2:**
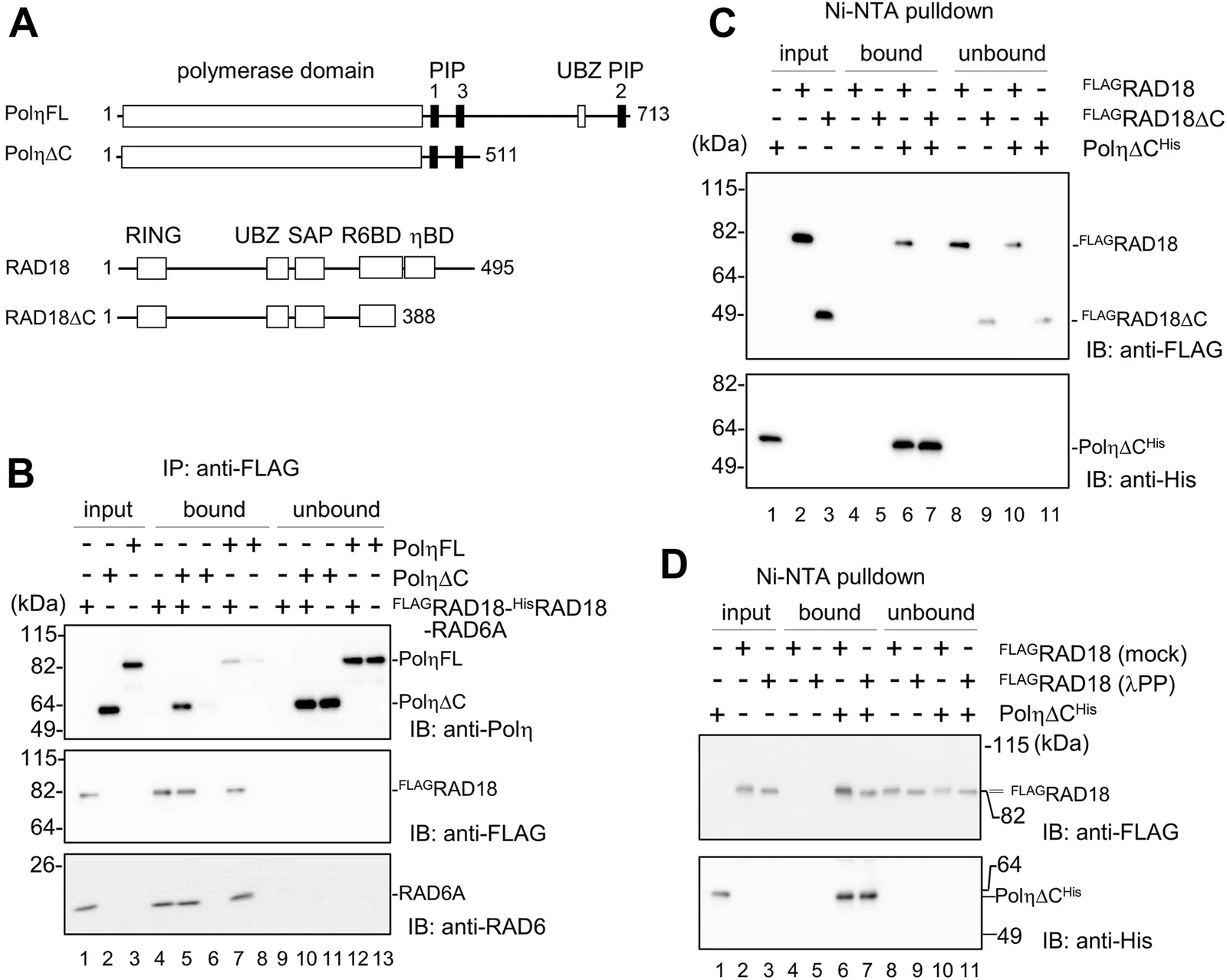
**N-terminus of human Polη directly binds RAD18 *in vitro*.***A,* schematic representations of the structures of Polη, RAD18, and their C-terminus deletion mutants. *B,* anti-FLAG immunoprecipitation assays. ^FLAG^RAD18-^His^RAD18-RAD6A was incubated with either untagged PolηFL or untagged PolηΔC, and immunoprecipitated with anti-FLAG antibody. Immunoprecipitated complexes were analyzed by Western blotting with the indicated antibodies. Lanes 1 to 3: 20% of RAD18 complex input, 10% of PolηFL input, and 10% of PolηΔC input used in immunoprecipitation, respectively; lanes 4 to 8: bound fractions; lanes 9 to 13: 10% of unbound fraction. *C,* Ni-NTA pull-down assays. PolηΔC^His^ was incubated with either ^FLAG^RAD18 or ^FLAG^RAD18ΔC, and pulled down with Ni-NTA magnetic beads. The complexes were analyzed by Western blotting with the indicated antibodies. Lanes 1, 2, and 3: 50% of PolηΔC input, 20% of RAD18 input, and 20% of RAD18ΔC input used in immunoprecipitation, respectively; lanes 4 to 7: bound fractions; lanes 8 to 11: 11% of unbound fraction. *D,*^FLAG^RAD18 was either mock-treated or treated with λPP and subjected to Ni-NTA pull-down assays as indicated in *(C)*. Lanes 1, 2, and 3: 30% of PolηΔC input, 10% of mock-treated RAD18 input, and 10% of λPP-treated RAD18 input used in immunoprecipitation, respectively; lanes 4 to 7: bound fractions; lanes 8 to 11: 10% of unbound fraction.

These findings demonstrate that the interaction between Polη and RAD18 occurs *via* two distinct modes in cells: one that requires ubiquitinated PCNA together with the C-terminal region of Polη for the direct interaction with RAD18 as reported previously in addition to the stimulation of PCNA ubiquitination, and the other representing a newly identified PCNA-independent interaction that is, to our surprise, mediated by the N-terminal domains of Polη. In the following analyses, we focused on characterizing this PCNA-independent mode of interaction.

### The N-terminus of Polη is crucial for its direct interaction with RAD18

To study the physical interaction between human Polη and RAD18, we performed *in vitro* immunoprecipitation assays with purified proteins produced in *E.coli*. The FLAG-tagged RAD18/His-tagged RAD18/RAD6A (^FLAG^RAD18-^His^RAD18-RAD6A) complex was incubated with either PolηFL or PolηΔC, and immunoprecipitated with anti-FLAG beads. As shown in Figures 2*B* and S2*A*, PolηΔC bound to the RAD18 complex as efficiently as PolηFL. Although the C-terminus of Polη had been reported to be responsible for the interaction between Polη and RAD18 (29), our results demonstrate for the first time that the N-terminal region of Polη can mediate this interaction.

Previous reports indicate that the C-terminal region of RAD18 (amino acids 402–444) and its phosphorylation are crucial for its interaction with Polη (36, 38). To further examine this, we purified C-terminally His-tagged PolηΔC (PolηΔC^His^), ^FLAG^RAD18, and ^FLAG^RAD18ΔC (amino acids 1–388; Fig. 2*A*) produced in insect cells. PolηΔC^His^ was incubated with either ^FLAG^RAD18 or ^FLAG^RAD18ΔC and pulled down with Ni-NTA magnetic beads. As shown in Figures 2*C* and S2*B*, ^FLAG^RAD18, but not ^FLAG^RAD18ΔC, bound to PolηΔC^His^.

To test whether RAD18 phosphorylation affects its affinity for the N-terminal region of Polη, ^FLAG^RAD18 was mock-treated or dephosphorylated with λ protein phosphatase (λPP) and subjected to the Ni-NTA pull-down assay (Figs. 2*D* and S2*C*). The λPP-treated ^FLAG^RAD18, which migrated faster than mock-treated ^FLAG^RAD18 on SDS-PAGE, bound to PolηΔC^His^ less efficiently than mock-treated ^FLAG^RAD18, indicating that phosphorylation of RAD18 enhances its ability to bind the N-terminal region of Polη. Together, these results suggest that the N-terminus of Polη interacts directly with RAD18 and that phosphorylation of RAD18 stimulates this interaction.

### The Polη-RAD18 complex is not formed on DNA

To examine the significance of the Polη-RAD18 interaction, we analyzed the DNA-binding properties and compared them with those of Polη and RAD18 alone. We previously demonstrated using an electrophoretic mobility shift assay (EMSA) with purified proteins that PolηΔC preferentially binds primer-template DNA, binds weakly to ssDNA, and binds poorly to dsDNA (13). In Figure S3, we examined the binding of the RAD18 complex to primer-template DNA, ssDNA, and dsDNA. When 5 fmol of primer-template DNA (^32^P-labeled 27-1/ND49) was incubated with more than 50 fmol of the ^FLAG^RAD18-^His^RAD18-RAD6A complex, a shifted band corresponding to a protein-DNA complex was observed (Fig. S3*A*). A CPD in the template strand located immediately downstream of the primer 3′ end did not affect the DNA-binding activity of the RAD18 complex (Fig. S3A, lanes 5–8). When ssDNA (30-1) was used as a substrate, no protein-DNA complex was detected (Fig. S3*B*). When dsDNA (30-1/ND30) was used, 200 fmol of the ^FLAG^RAD18-^His^RAD18-RAD6A complex was required to observe a detectable protein-DNA complex (Fig. S3*C*). Together with a previous report showing that the (RAD18)_2_-^His^RAD6B complex binds more efficiently to forked DNA than to ssDNA or dsDNA (45), these results indicate that the RAD18 complex preferentially binds to DNA substrate containing both single-stranded and double-stranded regions, such as primer-template DNA.

To examine whether Polη and RAD18 influence each other’s DNA-binding activity, increasing amounts of ^His^PolηΔC (2.5, 5, 10, and 20 fmol) were titrated in the presence or absence of 10 fmol of the (^His^RAD18)_2_-RAD6A complex (Fig. 3*A*). A PolηΔC-DNA complex was detected with as little as 2.5 fmol of PolηΔC in the absence of the RAD18 complex (Fig. 3*A*, lanes 2 and 12), whereas 50 fmol of the RAD18 complex was required to detect a RAD18-DNA complex (Fig. S3*A*). This indicates that the RAD18 complex has a much lower affinity for primer-template DNA than PolηΔC. Although the representative gel appears to suggest a modest increase in DNA binding in the presence of RAD6–RAD18, quantitative analysis of five independent experiments revealed only minor and inconsistent differences between samples with and without RAD6-RAD18 (Fig. S4). These results indicate that RAD6–RAD18 does not measurably alter the DNA-binding affinity of PolηΔC under the conditions tested. We did not detect any significant change in the DNA-binding affinity of either PolηΔC or the RAD18 complex in the presence of the other, regardless of whether the primer-template DNA contained a CPD (Fig. 3*A*).

**Figure 3:**
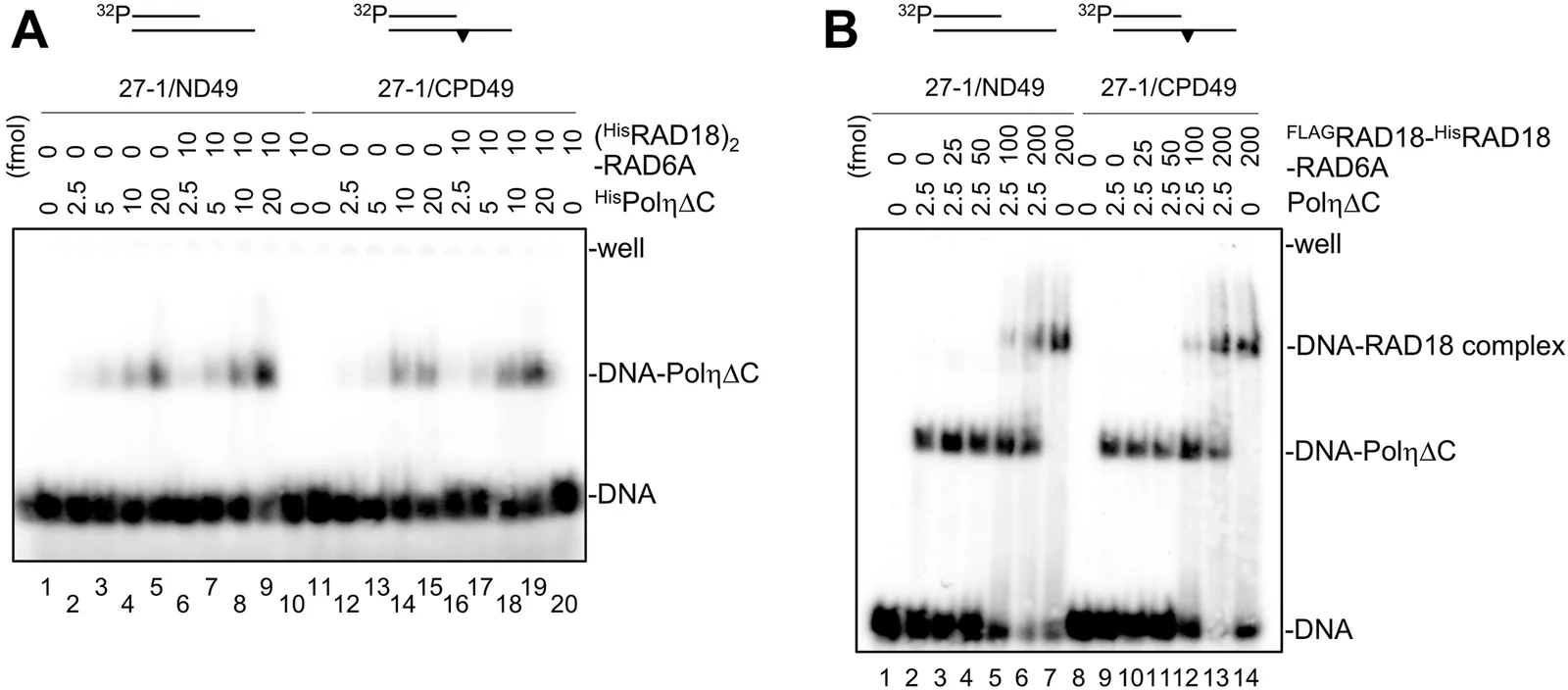
**The PolηΔC-RAD18 complex is not formed on DNA.** Electrophoretic mobility shift assay. *A,* increasing amounts of ^His^PolηΔC (2.5, 5, 10, and 20 fmol) were incubated with ^32^P-labeled 27-1/ND49 or 27-1/CPD49 (5 fmol) in the presence or absence of (^His^RAD18)_2_-RAD6A (10 fmol). The resulting DNA-protein complexes were separated by PAGE. *B,* untagged PolηΔC (2.5 fmol) was incubated with ^32^P-labeled 27-1/ND49 or 27-1/CPD49 (5 fmol) in the presence of increasing amounts of ^FLAG^RAD18-^His^RAD18-RAD6A (25, 50, 100, and 200 fmol). PolηΔC (2.5 fmol) alone and ^FLAG^RAD18-^His^RAD18-RAD6A (200 fmol) alone were incubated with the substrate in lanes 2 and 9, and in lanes 7 and 14, respectively.

Next, we titrated higher amounts of the ^FLAG^RAD18-^His^RAD18-RAD6A complex into binding reactions containing PolηΔC and either 27-1/ND49 or 27-1/CPD49 DNA (Fig. 3*B*). When 100 or 200 fmol of the RAD18 complex was added to reactions containing 2.5 fmol of PolηΔC, two shifted bands were observed (Fig. 3*B,* lanes 5, 6, 12, and 13). One band corresponded to the PolηΔC-DNA complex (as in lanes 2 and 9), and the other corresponded to the RAD18-RAD6-DNA complex (as in lanes 7 and 14). A shifted band corresponding to a ternary PolηΔC-RAD18-RAD6-DNA complex was not detected. Instead, upon increasing the RAD18 complex from 100 to 200 fmol, the intensity of the PolηΔC-DNA band decreased, whereas the intensity of the RAD18-RAD6-DNA band increased (Fig. 3*B,* lanes 5, 6, 12 and 13). These observations indicate that the RAD18-RAD6 complex and PolηΔC do not preferentially bind simultaneously and stably to the same primer-template DNA molecule, regardless of the presence of the CPD. However, at this stage we cannot exclude the possibility that a low abundance ternary complex exists but remains undetectable in the EMSA due to its scarcity in the reaction mixture (Fig. 2*B*).

### Binding of Polη to DNA interferes with the Polη-RAD18 interaction

To further examine the interactions among Polη, the RAD18-RAD6 complex, and DNA, we performed immunoprecipitation assays with purified proteins in the presence of DNA. The ^FLAG^RAD18-^His^RAD18-RAD6A complex was incubated with either PolηFL or PolηΔC in the presence of anti-FLAG beads and increasing amounts of either 5′-protruding DNA (14-2/ND30-2) or 3′-protruding DNA (16-2/ND30-2). Notably, the former can serve as a primer-template for DNA polymerases, whereas the latter cannot.

PolηFL bound to the RAD18 complex in the absence of DNA (Fig. 4*A,* lane 4). Quantitative analysis demonstrated that the presence of DNA significantly reduced the interaction between Polη and the RAD18 complex in a DNA-dose-dependent manner (Fig. 4*A,* lanes 5–7 and 8–10). To our knowledge, this is the first demonstration that DNA interferes with the interaction between Polη and RAD18.

**Figure 4:**
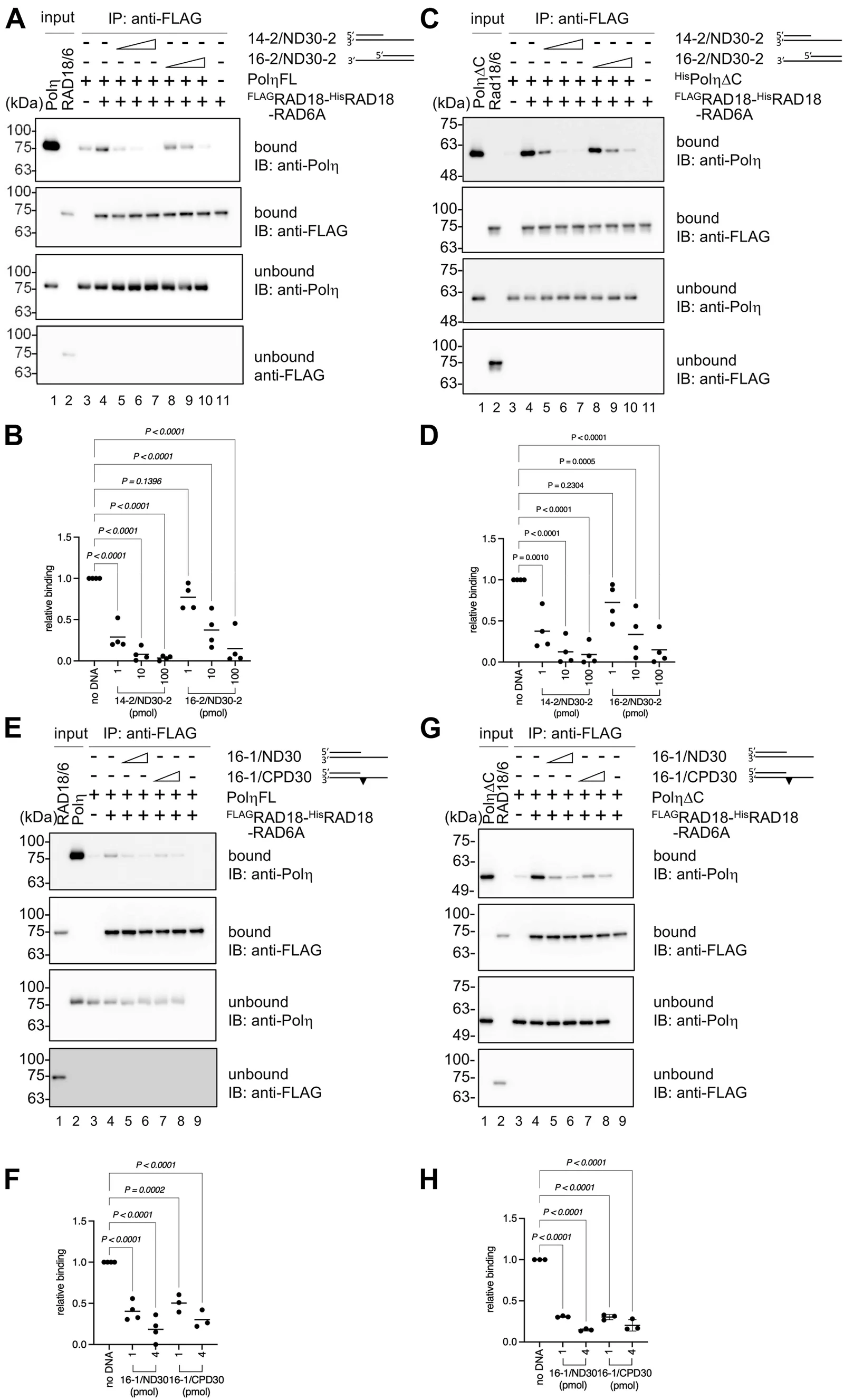
**DNA inhibits the interaction between Polη and RAD18.***A–H,* anti-FLAG immunoprecipitation. ^FLAG^RAD18-^His^RAD18-RAD6A was incubated with either untagged PolηFL or untagged PolηΔC (^His^PolηΔC in C) in the presence of 1, 10, or 100 pmol of 5′-protruding (14-2/ND30-2) or 3′-protruding (16-2/ND30-2) DNA *(A and C)* or in the presence of 1 or 4 pmol of 16-1/ND30 or 16-1/CPD30 DNA *(E and G)*, and analyzed as in Figure 2*B*. *A, E, and G,* lanes 1 and 2: 10% of PolηFL or PolηΔC input and 20% of RAD18 complex input used in immunoprecipitation, respectively. *C,* lanes 1 and 2: 10% of PolηΔC input and 50% of RAD18 complex input used in immunoprecipitation, respectively. Lanes 3 to 11 in *(A and C)* and lanes 3 to 9 in *(E and G)*: 10% of unbound fraction or bound fraction, as indicated. *B, D, F, and H,* PolηFL or PolηΔC in the immunoprecipitated complexes in *A, C, E, and G* were quantified and expressed as a ratio to that in assays without DNA. The experiment was repeated at least three times, and individual values were plotted. Data are presented as the mean ± S.D. Statistical analysis was performed using one-way ANOVA, followed by Dunnett’s multiple-comparisons test (*panel B*, *F* = 26.79, *p* < 0.0001; *panel D*, *F* = 12.44, *p* < 0.0001; *panel F*, *F* = 33.17, *p* < 0.0001; *panel H*, *F* = 299.4, *p* < 0.0001). Adjusted *p* values are shown.

Among the DNA substrates tested, the 5′-protruding DNA (14-2/ND30-2) inhibited the interaction more strongly than the 3′-protruding DNA (16-2/ND30-2), as shown in Figure 4, *A* and *B*. Similar results were obtained when PolηΔC was used instead of PolηFL (Fig. 4*C* and *D*). The CPD-containing primer-template (16-1/CPD30) interfered with the interaction between PolηFL and the RAD18 complex as efficiently as the undamaged primer-template (16-1/ND30) (Fig. 4*E* and *F*), and the same was observed with PolηΔC (Fig. 4*G* and *H*). Based on these findings, we considered the possibility that DNA binding and RAD18 binding to Polη are mutually exclusive.

### Overlapping binding sites for DNA and RAD18 on Polη

Although both Polη and RAD18 can bind primer-template DNA irrespective of the presence of a CPD, the RAD18 complex exhibits much weaker affinity for the primer-template DNA than PolηΔC (Figs. 3*A*, *B*, and S3*A*). We therefore examined the possibility that the interaction between Polη and the RAD18 complex is diminished when Polη binds to DNA. To this end, we mutated Lys323 in PolηΔC, a basic residue that interacts with template DNA (40), to an acidic residue (K323E), and purified the corresponding mutant protein, ^His^PolηΔC(K323E). This mutation reduced the DNA-binding activity of PolηΔC by approximately two-fold (Fig. S5*A*–*C*), as well as its DNA polymerase activity (Fig. S5*D*).

In immunoprecipitation assays, the amount of ^His^PolηΔC(K323E) bound to the RAD18 complex in the absence of DNA was lower than that of the WT ^His^PolηΔC (Fig. 5*A*, lanes 5 and 10), suggesting that Lys323 contributes not only to DNA binding but also to the interaction with RAD18. In addition, the interaction between PolηΔC(K323E) and the RAD18 complex was less susceptible to inhibition by the 5′-protruding DNA than the interaction between WT PolηΔC and the RAD18 complex (Fig. 5*A*, lanes 6–8 and 11–13, and 5B). The same tendency was observed with the full-length mutant ^His^PolηFL(K323E) (Fig. S6*A*–*C*), although the effect was modest.

**Figure 5:**
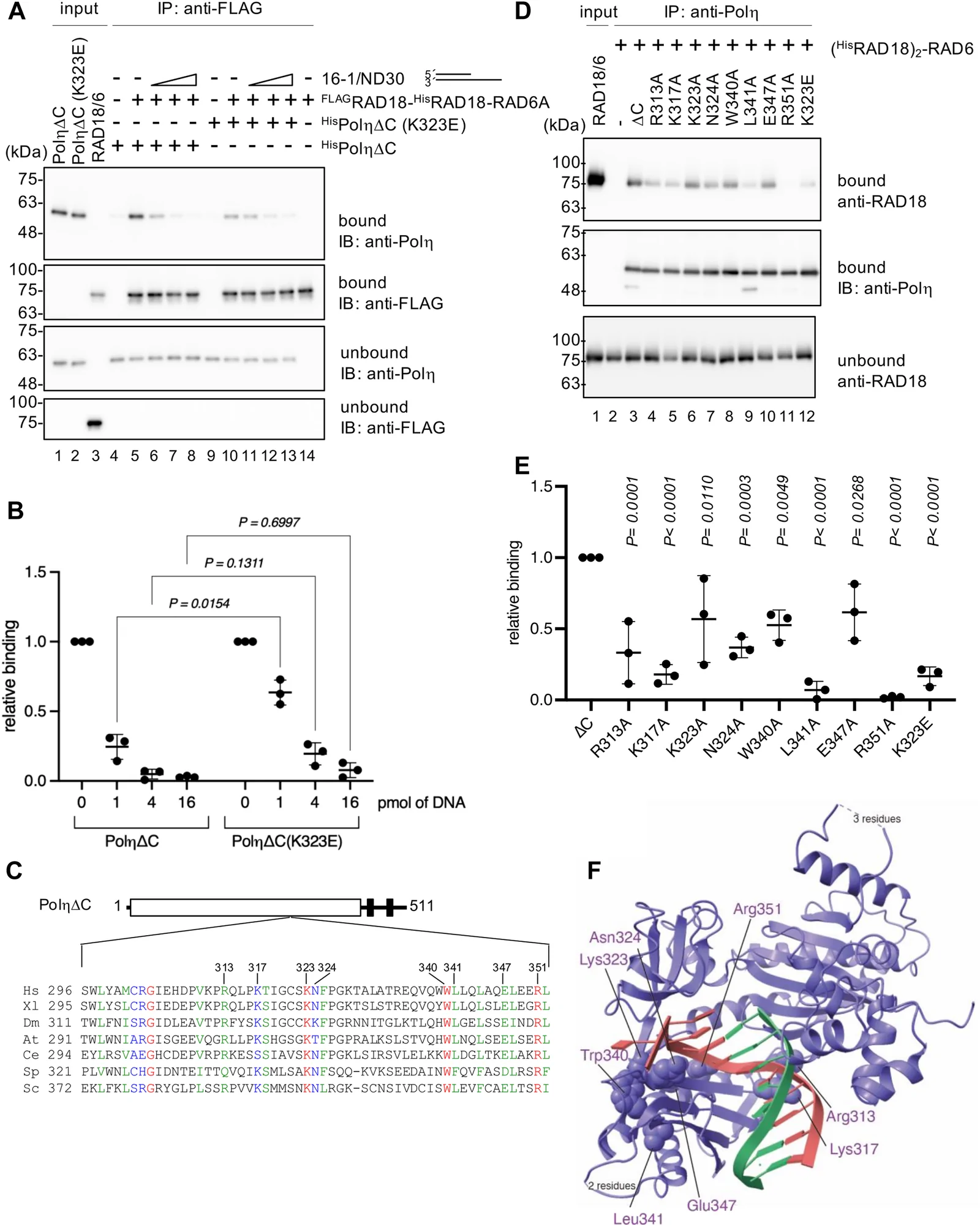
**RAD18 binding activities of PolηΔC DNA-binding mutants.***A,* anti-FLAG immunoprecipitation. ^FLAG^RAD18-^His^RAD18-RAD6A was incubated with ^His^PolηΔC or ^His^PolηΔC(K323E) in the presence of 0, 1, 10, or 100 pmol of 5′-protruding DNA (16-1/ND30), and analyzed as in Figure 2*B*. Lanes 1, 2, and 3: 10% of PolηΔC, PolηΔC(K323E), and 20% of RAD18 input used in immunoprecipitation, respectively. *B,* PolηΔC or PolηΔC(K323E) in the immunoprecipitated complexes was quantified and expressed as a ratio to that in assays without DNA. The experiment was repeated three times and individual values were plotted. Individual values were plotted and the means ± SDs of three biologically independent experiments are shown. Statistical analysis was performed using RM two-way ANOVA, with DNA concentration and Polη genotype as factors and both factors matched. Significant effects were observed for DNA concentration (*F*_1.032, 2.064_ = 391.3, *p* = 0.0022), Polη genotype (*F*_1.000, 2.000_ = 71.88, *p* = 0.0136), and the DNA concentration × Polη genotype interaction (F_1.588, 3.177_ = 77.35, *p* = 0.0021). Pairwise comparisons between PolηΔC and PolηΔC(K323E) at each DNA concentration were performed using Šídák’s multiple-comparisons test; adjusted *p* values are indicated. *C,* amino acid sequence alignment of Polη orthologs (46).Letters in *red, green, and blue* indicate degrees of conservation ranging from high to modest. *D,* anti-Polη immunoprecipitation. (^His^RAD18)_2_-RAD6A was incubated with either PolηΔC^His^ or its point mutant bound to Protein G conjugated with anti-Polη. Immunoprecipitated complexes were analyzed by Western blotting with the indicated antibodies. Lane 1, 10% of RAD18 input used in immunoprecipitation. *E,* RAD18 in the immunoprecipitated complex (lanes 3–12 in *D*) were quantified, corrected for the amount of each PolηΔC mutant and expressed as a ratio to that of PolηΔC. Individual values were plotted and the means ± SDs of three independent experiments are shown. Statistical analysis was conducted by one-way ANOVA (*F* = 12.89, *p* < 0.0001), followed by Dunnett’s multiple comparisons test, comparing each group to the control, WT PolηΔC). Exact adjusted *p* values for pairwise comparisons are indicated. *F,* crystal structure of the DNA–Polη(1-432) complex (PDB ID: 3MR2) (46). Amino acids mutated in this study are presented.

To identify residues in Polη that contribute to binding RAD18 but not DNA, we introduced several mutations around K323 in PolηΔC^His^ (Fig. 5*C* and *F*), purified the mutant proteins, and examined their RAD18-binding, DNA-binding, and DNA polymerase activities (Fig. S7). In coimmunoprecipitation assays using each PolηΔC^His^ mutant and the (^His^RAD18)_2_-RAD6A complex, the anti-Polη antibody immunoprecipitated significantly less RAD18 than with WT PolηΔC^His^, regardless of the mutant tested (Fig. 5*D* and *E*). In particular, RAD18 binding to the K317A, L341A, and R351A mutants, as well as to the K323E mutant, was reduced by approximately 80% compared with WT PolηΔC^His^. Among these mutants, R351A exhibited a marked decrease in DNA binding, whereas K317A and L341A showed only partial, non-significant reductions (Fig. S7*B* and *C*). The DNA polymerase activities of L341A and R351A tended to be lower than that of PolηΔC when assayed on singly-primed M13 DNA (Fig. S7*D*), although both mutants were still able to bypass CPD lesions on the short primer-template substrate (Fig. S7*E*). Notably, the K317A mutant displayed DNA polymerase activity comparable to that of PolηΔC across both assays (Fig. S7*D* and *E*). Based on these observations, we considered K317A to be a mutant whose interaction with RAD18 is much more severely impaired than its interaction with DNA, and therefore selected it for further characterization in cells.

AlphaFold 3 was used to model the association of RAD18 with PolηΔC. To first assess the reliability of the prediction, we generated an AlphaFold 3 model of the DNA-PolηΔC complex and compared it with the published crystal structure (46). The model closely recapitulated the crystal structure (Figs. 5*F* and S8*A*), and the predicted local distance difference test scores at the DNA-PolηΔC interface were relatively high (Fig. S8*B*), indicating that AlphaFold 3 reliability predicts the DNA-PolηΔC complex. We then generated an AlphaFold 3 model of the RAD18-PolηΔC complex (Fig. S8*C* and *D*). The model suggests that two α-helics of RAD18 (residues 435–441 and 444–464) are positioned over the DNA-binding surface of Polη (Fig. S8*C* and *E*), although the predicted local distance difference test scores for these helics are relatively low (Fig. S8*D*). Among the Polη residues examined in this study, Arg313 and Lys317 of PolηΔC are predicted to interact with Trp457 of RAD18, whereas Asn324 of PolηΔC is predicted to interact with Asp445 of RAD18 (Fig. S8*E*). These observations support our biochemical findings that the DNA- and RAD18-binding surfaces on Polη overlap, and that binding of DNA and RAD18 to Polη is mutually exclusive.

### The Polη-RAD18 interaction contributes to cellular UV resistance

To investigate the cellular functions of the RAD18-binding site of Polη *in vivo*, we examined whether Polη mutants could complement the UV sensitivity of Polη-deficient XP-V cells. For this purpose, we generated XP2SASV3 cell populations stably expressing FLAG-tagged PolηFL, PolηΔC, or the K317A mutants (Fig. S9*A*). To avoid potential biases arising from clonal variability, we analyzed bulk populations expressing each exogenous gene. The observed differences in UV tolerance were not accompanied by obvious differences in protein expression between the corresponding WT and K317A constructs (Fig. S9). As shown in Figure 6, the PolηFL(K317A) mutant was less effective at complementing the UV sensitivity of XP-V cells than PolηFL (LD_50_ values of 5.46 and 6.41 J/m^2^, respectively; 0.852 *versus* 1). Polη contains a UBZ domain and three PIP motifs, PIP1, PIP3, and PIP2 in order from the N-terminus, that function redundantly to promote cellular survival after UV irradiation. To explore the relationship between Lys317 and these domains, we analyzed PolηΔC, which retains PIP1 and PIP3 but lacks the UBZ domain and PIP2 (Fig. 2*A*) (32). Consistent with the absence of these two domains, PolηΔC complemented UV sensitivity less efficiently than PolηFL (LD_50_ values were 5.35 and 6.41 J/m^2^, respectively). Importantly, the PolηΔC(K317A) mutant was significantly less effective than PolηΔC in rescuing UV sensitivity (LD_50_ values of 4.38 and 5.35 J/m^2^; 0.819 *versus* 1), indicating that Lys317 plays a critical role in UV survival in the PolηΔC background. These findings also suggest that the functions of Lys317 are distinct from those of UBZ and PIP2.

**Figure 6:**
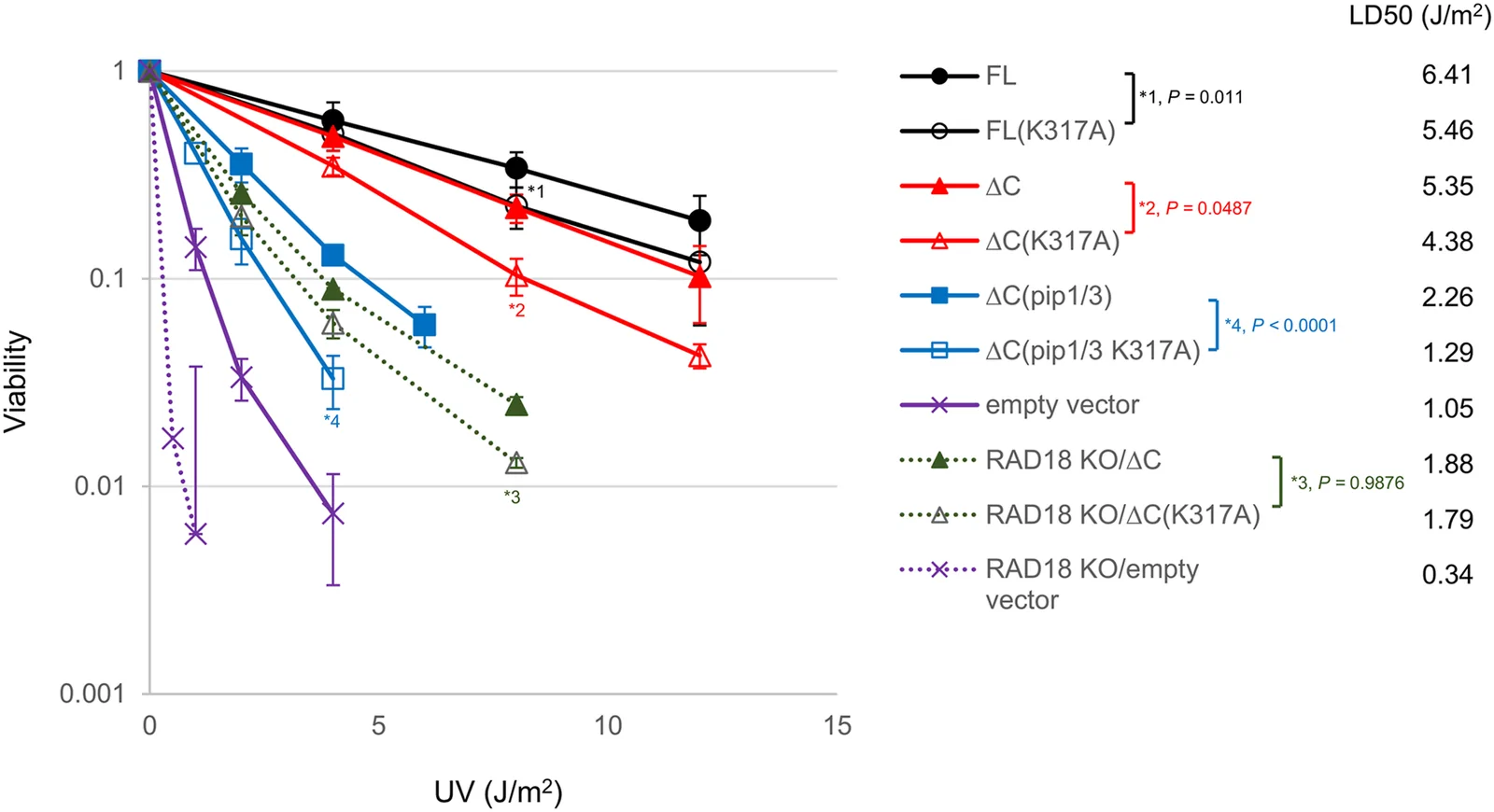
**Cellular functions of Lys317 of Polη.** UV sensitivities of cells. XP-V cells *(solid lines)* stably expressing PolηFL, PolηΔC, RAD18 binding mutants of PolηFL(K317A) and PolηΔC(K317A), the PCNA-binding mutant of PolηΔC (pip1/3), RAD18-binding and PCNA-binding mutant of PolηΔC (pip1/3 K317A), or containing the empty vector, and XP-V/RAD18 KO cells *(dotted lines)* stably expressing PolηΔC, PolηΔC(K317A), or containing the empty vector were used. The cells were irradiated with UV, and their viabilities were measured. Means ± SDs of at least three independent experiments are plotted. Statistical analyses were performed using a one-way ANOVA followed by Šídák’s multiple-comparisons test for the data at 8 J/m^2^ (*F* = 28.06, *p* < 0.0001), and a two-tailed, unpaired *t* test for the data at 4 J/m^2^ comparing PolηΔC (pip1/3) and PolηΔC (pip1/3 K317A). Exact *p* values are indicated. LD50 values are shown on the *right*.

We next examined the role of Lys317 in the PolηΔC(pip1/3) background, in which PIP1 and PIP3 are inactivated by F443A/L444A/F483A/F484A mutations (Fig. S9*C*). PolηΔC(pip1/3) complemented the UV sensitivity of XP-V cells, although less efficiently than PolηΔC, consistent with its impaired PCNA-binding (35). Introduction of the K317A mutation into PolηΔC(pip1/3) markedly increased UV sensitivity (LD_50_ decreased from 2.26 to 1.29 J/m^2^; 1 *versus*. 0.571), again demonstrating that Lys317 contributes to survival after UV irradiation. The substantial impact of K317A in this background likely reflects combined defects in both DNA binding and RAD18 binding. These results further indicate that the functions of Lys317 are distinct from those of PIP1 and PIP3. To determine whether the cellular phenotype of the K317A mutant is associated with impaired RAD18 binding, we performed co-immunoprecipitation analyses using cell extracts. The K317A mutation reduced the interaction between Pol η and RAD18 in both the full-length and ΔC proteins (Fig. S10*A*–*D*), consistent with the *in vitro* binding data. These observations indicate that the N-terminal RAD18-interaction interface contributes to Pol η–RAD18 association in cells and support its functional relevance for UV-damage tolerance.

To determine whether the reduced complementation efficiency of PolηΔC(K317A) was due to weakened interaction with RAD18, we performed complementation assays in a RAD18-deficient background. We established an XP-V/RAD18-KO cell line stably expressing either ^FLAG^PolηΔC or ^FLAG^PolηΔC(K317A) (Fig. S9*B*). As shown in Figure 6, PolηΔC(K317A) complemented the UV sensitivity of XP-V/RAD18 KO cells slightly less effectively than PolηΔC (LD_50_ values of 1.79 and 1.88 J/m^2^, respectively; 0.952 *versus* 1). Strikingly, the difference between PolηΔC and PolηΔC(K317A) was smallest in the RAD18-deficient background. This pattern indicates that most of the Lys317-dependent contribution to UV survival is RAD18-dependent, while the smaller residual defect in the RAD18-KO background likely reflects the partial reduction in DNA-binding activity caused by the K317A mutation. Collectively, these findings suggest that the interaction between the N-terminal region of Polη and RAD18 contributes significantly to cellular viability after UV irradiation.

## Discussion

Translesion DNA synthesis (TLS) by Polη critically depends on RAD18-mediated monoubiquitination of PCNA, and Polη itself enhances this ubiquitination step, establishing a mutually reinforcing relationship among Polη, RAD18, and PCNA that has been recognized as a central regulatory axis in TLS. Despite extensive studies on this tri-partite module, the mechanistic details of how Polη is recruited and activated remain incompletely understood. In this study, we identified a previously unappreciated interaction between Polη and RAD18 that occurs independently of PCNA ubiquitination, promoting us to investigate its biochemical properties and physiological relevance.

We demonstrated that the N-terminal region of human Polη directly interacts with RAD18 and that RAD18 competes with DNA for binding to Polη *in vitro*, owing to the overlap between the Polη domains responsible for RAD18 binding and those responsible for DNA binding. In cellular experiments, the PolηΔC(K317A) mutant, defective in RAD18 binding but proficient in DNA binding and DNA polymerase activity, was less efficient than PolηΔC at complementing the UV sensitivity of Polη-deficient cells in the presence of RAD18. This finding indicates that RAD18 binding through the N-terminus of Polη contributes to cellular survival after UV irradiation. Together, our results not only support a model in which the Polη-RAD18 interaction is required for TLS activation, but also provide, for the first time, mechanistic insight into a RAD18-dependent recruitment process in which Polη transitions from binding to RAD18 to binding primer-template DNA.

### Physical interaction between the N-terminus of human Polη and RAD18

The C-terminal region of human Polη (amino acids 556–713) has previously been reported to interact with RAD18 (29). In contrast to this prevailing view, we found that the N-terminal region of Polη, spanning amino acids 1 to 511, also interacts with RAD18 (Fig. 2), and we identified several residues within the 313 to 351 region that are critical for this interaction (Fig. 5). Previous studies have shown that the C-terminal region of RAD18 (amino acids 401–445) binds to the C-terminal region of Polη (29), and that phosphorylation of RAD18 at S409 and within the S-box (amino acids 432–444) enhances its interaction with full-length Polη *in vivo*. In agreement with these findings, we observed that the RAD18 phosphorylation promotes its interaction with PolηΔC *in vitro* (Fig. 2*D*). Furthermore, binding of both full-length Polη and PolηΔC to RAD18 was inhibited by DNA (Fig. 4).

Previous studies reporting C-terminal Polη-RAD18 interactions largely relied on GST-fused Polη fragments and pull-down assays using cell lysates. We do not intend to challenge the possibility that the Polη C terminus can interact with RAD18; such interactions may indeed occur in cells, where additional proteins, posttranslational modifications, or multicomponent assemblies could stabilize or bridge contacts among Polη, RAD18, PCNA, and other factors. These considerations may be important for understanding how Polη-RAD18 interactions are organized *in vivo*. By contrast, our analyses used purified proteins without GST fusion and Polη fragments with domain boundaries that differ slightly from those examined previously, conditions that selectively detect direct and relatively stable interactions. These methodological differences likely explain why we did not detect binding between the isolated Polη C-terminal region and RAD18 in our reconstituted system (data not shown), while allowing us to identify a direct interaction mediated by the Polη N-terminus.

Although both the N- and C-terminal regions of Polη may contribute to RAD18 binding *in vivo*, our results clearly demonstrate that the N-terminus of Polη is capable of mediating a direct physical interaction with RAD18. The C-terminal region of Polη contains PIP2, which interacts with PCNA to enhance RAD18-mediated monoubiquitination of PCNA, and a UBZ domain that binds monoubiquitinated PCNA, both of which contribute to Polη nuclear foci formation after UV irradiation. In contrast, the functional relevance of protein interactions mediated by the N-terminal region has been far less explored. Several proteins have been shown to interact with Polη and for some of these interactions, notably those with PCNA and REV1, the relevant domains and critical amino acid residues have been identified (35, 36, 47), enabling mechanistic analyses using point mutant approaches. However, the critical amino acids required for interactions between the catalytic N-terminal domain of Polη and other partners, including the mismatch repair protein MLH1 (48) and E3 ubiquitin ligase Pirh2 (49) have yet to be identified.

### Overlapping of DNA-binding and RAD18-binding domains in Polη

The Polη-RAD18 interaction has been reported to contribute to the mutual loading of these proteins onto chromatin in cells (34). However, in our study, neither protein enhanced the loading of the other onto DNA, and simultaneous loading of both proteins onto the same DNA molecule was rarely observed *in vitro* (Fig. 2). Instead, the interaction between Polη and RAD18 was disrupted by DNA, particularly by primer-template DNA that serves as a substrate for DNA polymerases (Fig. 4). The Polη K323E mutant exhibited not only reduced DNA-binding activity but also diminished RAD18-binding activity. Importantly, the remaining Polη-RAD18 interaction in the K323E mutant was markedly more resistant to inhibition by DNA than that of WT Polη (Fig. 5), suggesting that the DNA-binding and RAD18 binding domains of Polη overlap. Consistent with this idea, all point mutations spanning the 313 to 351 amino-acid region, which contains the DNA-binding domain of Polη, reduced Polη-RAD18 binding (Fig. 5). Although we cannot exclude the possibility that additional residues outside this region contribute to RAD18 binding, our results indicate that the DNA-binding and RAD18-binding domains of Polη largely overlap.

Consistent with our biochemical analyses, AlphaFold 3 modeling of the PolηΔC-RAD18 complex suggests that the predicted RAD18-binding surface on PolηΔC lies directly over the DNA-binding region. Although the confidence for the RAD18 helices in the model was relatively low, the overall alignment is compatible with our mutational data showing that residues within the 313 to 351 region contribute to both DNA binding and RAD18 binding. This spatial arrangement provides a plausible structural basis for why DNA and RAD18 compete for an overlapping interface on Polη. While the AlphaFold prediction should be interpreted cautiously, they support a model in which Polη cannot engage RAD18 and DNA simultaneously in a stable manner, reinforcing the mutually exclusive relationship inferred from our biochemical and cellular findings.

The DNA substrate used in this study minimizes, but does not eliminate, the possibility that Polη and RAD18–RAD6 bind independently to different regions of the same DNA molecule. Such independent binding would complicate interpretation of whether the observed effects reflect competition between DNA binding and Pol η–RAD18 interaction. Nevertheless, variations in DNA length and structure may alter the relative binding properties of Pol η and RAD18–RAD6. More complex DNA substrates may therefore reveal additional regulatory features, although interpretation of such experiments may be complicated by the availability of multiple independent binding sites. Future studies using a wider range of DNA structures will be required to address this issue.

### Identification of a RAD18-binding-specific mutant of Polη

Among the point mutants analyzed, K317A, L341A, and R351A exhibited defects in RAD18 binding that were comparable to or more severe than the defect observed in the K323E mutant (Fig. 5*D* and *E*). The DNA-binding activity of R351A was markedly reduced, whereas the K317A and L341A mutants showed only mild and statistically insignificant reductions compared with WT Polη (Fig. S7*B* and *C*). The L341A mutant also displayed reduced DNA-polymerase activity when singly primed M13 DNA was used as a template (Fig. S7*D*), but not when short oligonucleotide primer-templates were used (Fig. S7*E*). Similar phenotypes were observed in the R313A and K323A mutants. A plausible explanation is that these mutations impair primer-end recognition in the presence of excess single-stranded M13 DNA, perhaps due to altered specificity toward ssDNA *versus* primer-template DNA. In contrast, the K317A mutant retained DNA-polymerase activity and DNA-binding activity capable to those of WT Polη, while its RAD18-binding activity was largely abolished. This profile indicates that K317A represents a RAD18-binding-specific separation-of-function mutant of Polη.

### Physiological relevance of the interaction between the N-terminus of Polη and RAD18

We demonstrated that Lys317 in Polη is important for cell survival after UV irradiation. Notably, its contribution to UV resistance became more pronounced in the absence of the C-terminal region of Polη (Fig. 6). One possible explanation is that the C-terminus of Polη may also be capable of binding RAD18 (29), although such an interaction was not detected in our reconstituted system as mentioned above. Polη contains three PIP motifs that have distinctive roles in stimulating DNA synthesis and enhancing RAD18-mediated PCNA monoubiquitination, yet function redundantly to promote cellular viability after UV exposure (35). In addition, Polη harbors two REV1-interacting motifs that redundantly mediate Polη-REV1 interaction (36, 37). Thus, Polη-mediated TLS and related DNA damage tolerance mechanisms appear to rely on multiple, partially redundant protein-protein interactions that together ensure robust UV survival.

It is also possible that interactions involving the C-terminal region of Polη, such as those with ubiquitinated PCNA or REV1, override or compensate for the loss of RAD18 binding caused by the K317A mutation. Furthermore, PolηΔC(K317A) was less effective than PolηΔC at rescuing the reduced viability of the pip1*/3* mutant (Fig. 6), suggesting that the Polη-RAD18 interaction and the Polη-PCNA interaction contribute to UV survival through distinct mechanisms. Consistent with this idea, the detrimental effect of K317A on UV survival was attenuated in a RAD18-deficient background (Fig. 6), indicating that Lys317 plays an important role in mediating the Polη-RAD18 interaction in cells exposed to UV irradiation. Collectively, these findings support the conclusion that the interaction between Polη and RAD18 mediated by Lys317 contributes significantly to cellular survival after UV irradiation.

### Two modes of the interaction between Polη and RAD18 in cells

Based on the biochemical and cellular data reported in this study, we propose a model for Polη recruitment to primer-template junctions (Fig. S11) and for the regulation of DNA damage tolerance mechanisms. When the replicative DNA polymerase encounters a lesion that blocks further DNA synthesis, the Polη-RAD18 complex accumulates near the stalled sites and promotes monoubiquitination of PCNA. Under these conditions, RAD18-dependent and PCNA-ubiquitination-dependent pathways, including TLS and template switching, are primed for activation in the replication factory. Among TLS polymerases, Polη is able to bypass a relatively broad spectrum of DNA lesions and, uniquely, can interact directly with RAD18. In addition, Polη promotes re-localization of REV1 and Polι to sites of Polη accumulation (36, 44, 50, 51) and can activate REV1- and Polκ-mediated DNA damage tolerance (52). Thus, among the various DNA damage tolerance pathways, Polη-mediated TLS is likely to be engaged first at stalled replication sites.

Although our working model emphasizes an early role for Polη at stalled replication forks, RAD18–RAD6-dependent lesion bypass can also occur during post-replicative repair after bulk DNA synthesis. In this context, the PCNA-independent interaction identified here may contribute not only to replication-coupled TLS but also to gap-filling events during late S or G2 phase. Furthermore, these mechanisms may function in parallel with or downstream of REV1-dependent TLS pathways depending on the cellular context. Once Polη is loaded onto the arrested primer-template junction, RAD18 is released from Polη, and Polη initiates TLS. In this scenario, TLS at CPDs may proceed in two steps. First PCNA monoubiquitination recruits the RAD18-Polη complex. Then, Polη dissociates from the RAD18 complex and is positioned at the primer terminus to catalyze lesion bypass. However, if Polη cannot bypass the lesion, it may readily dissociate from the primer-template DNA, thereby allowing other TLS polymerases to access the stalled replication sites through their interactions with monoubiquitinated PCNA and/or RAD18. This dynamic exchange among TLS polymerases ensures that multiple, partially redundant mechanisms can respond efficiently to replication-blocking lesions.

## Experimental procedure

### Proteins

Untagged Polη (31), untagged PolηΔC (35), hexahistidine tagged Polη at its N-terminus (^His^Polη) (53), His tagged Polη at its C-terminus (Polη^His^) (8), PolηΔC^His^ used in Figure 2*C* and *D* (13), ^FLAG^RAD18-^His^RAD18-RAD6A complex (20), and (^His^RAD18)_2_-RAD6A complex (20) were purified as described previously. PolηΔC^His^ and its point mutants (R313A, K317A, K323A, N324A, W340A, L341A, E347A, R351A, K323E) used in Figures 5*D*, *E*, and S7*A*–*E* were overproduced in BL21 (DE3) and purified as described previously (46).

For purification of ^His^PolηΔC, ^His^PolηΔC(K323E), and ^His^PolηFL(K323E), BL21 (DE3) harboring pET15-POLHΔC, pET28-POLHΔC(K323E), or pET15-POLH(K323E) was grown and cell lysates were prepared as described previously for ^His^Polη (53). Proteins were purified by sequential chromatography on Ni^2+^-charged HiTrap Chelating HP, HiTrap Heparin, and Superdex 200 columns (GE Healthcare). The peak fraction containing ^His^PolηΔC, ^His^PolηΔC(K323E), or ^His^PolηFL(K323E) was frozen in liquid nitrogen and stored at −80 °C.

To construct the FLAG-tagged human RAD18 expression plasmid in Sf9 cells, a FLAG-RAD18 cDNA fragment was obtained from a pAC-RAD6A/flagRAD18 construct (20) and subcloned into pFastBac Dual (Thermo Fisher Scientific). ^FLAG^RAD18 and ^FLAG^RAD18ΔC were expressed in Sf9 insect cells using the baculovirus expression system according to manufacturer’s protocols. Cells were collected by low-speed centrifugation and washed twice with ice-cold phosphate-buffered saline. The cell pellets were suspended six times the packed cell volume of ice-cold buffer A (20 mM sodium phosphate pH 7.2, 10% glycerol, 10 mM β-mercaptoethanol) containing 0.3 M NaCl, 1% NP-40, 0.5 mM phenylmethylsulfonyl fluoride, 50 μM EGTA, 0.4 μg/ml antipain, 0.4 μg/ml aprotinin, 0.1 μg/ml leupeptin, and 80 ng/ml pepstain A, and then incubated on ice for 15 min with occasional agitation. The suspension was centrifuged at 12,000*g* for 40 min. The supernatant was applied to a 5-mL HiTrap DEAE column (GE Healthcare) equilibrated with buffer A containing 0.3 M NaCl. The flow-through fraction was applied to ANTI-FLAG M2 Affinity Gel (Sigma-Aldrich) equilibrated with buffer A containing 0.3 M NaCl. The gel was washed with the equilibration buffer, and protein was eluted with buffer A containing 0.3 M NaCl and 100 μg/ml of FLAG peptide (Sigma-Aldrich). The eluted fraction was diluted with buffer A to 0.25 M NaCl, and then applied to a 1-mL HiTrap Heparin HP column (GE Healthcare) equilibrated with buffer A containing 0.25 M NaCl. The column was washed with the equilibration buffer, and proteins were eluted with a linear gradient of 250 to 800 mM NaCl in buffer A. The peak fraction containing ^FLAG^RAD18 or ^FLAG^RAD18ΔC was frozen in liquid nitrogen and stored at −80 °C.

### Antibodies

Monoclonal anti-FLAG M2-Peroxidase (A8592; Sigma-Aldrich), monoclonal anti-His-tag (D291; Medical and Biological Laboratories), polyclonal anti-RAD18 (70-023; BioAcademia), polyclonal anti-RAD6 (70-020; BioAcademia), anti-PCNA (PC10; Santa Cruz Biotechnology), anti-actin (sc-1616; Santa Cruz Biotechnology), and anti-Lamin B1 (12987-1-AP; Proteintech, Rosemont, IL) antibodies were purchased. Mouse monoclonal cl.5H10 and cl.5C6 antibodies against human Polη were generated as described previously (36). Rabbit polyclonal antibody against human Polη (anti-Polη133) was generated using a peptide containing amino acids 133 to 152 of human Polη as an antigen.

### Cell lines and cultures

SV-40 immortalized XP-V fibroblasts (XP2SASV3) and SV-40 immortalized normal human fibroblasts (WI38VA13) (43) were maintained in Dulbecco’s modified Eagle’s medium (DMEM) supplemented with 10% fetal bovine serum, 0.584 g/L L-glutamine, 0.07 g/L penicillin, and 0.15 g/L streptomycin. Cells were confirmed to be free of *mycoplasma* contamination.

*RAD18* KO XP2SASV3 cells (XP2SASV3/RAD18 KO) were established using CRISPR-Cas9 technology. A guide RNA coding sequence (5′-CCACACCGCAGCAAATCATCTAT-3′) was cloned into the pX459 vector (Addgene plasmid #62988) (54, 55). The designated plasmid was transfected into XP2SASV3 cells using the Neon transfection system (Thermo Fisher Scientific). Cells were selected for 24 h with puromycin (3 μg/ml). Single clones were isolated by limiting dilution. KO of *RAD18* gene was confirmed by DNA sequencing of the target region and the absence of the RAD18 protein was confirmed by immunoblotting.

pIRESneo2 carrying FLAG-POLH cDNA (pIRESneo2-FLAG-Polη) was transfected into XP2SASV3 cells to obtain FLAG-Polη-expressing cells using the Neon transfection system (Thermo Fisher Scientific) (35). XP2SASV3 and XP2SASV3/RAD18 KO cells expressing ^FLAG^PolηΔC, which lacks PIP2, UBZ, and the nuclear localization signal and is fused with an artificial nuclear localization signal at its C-terminus (35), were also established.

### Anti-FLAG immunoprecipitation from cell extracts

For Figure 1*A*, XP2SASV3 cells stably expressing exogenous PCNA or the K164R mutant (43) were transfected with siRNA targeting endogenous PCNA (5′-UAUGGUAACAGCUUCCUCCdTdT-3′) using DharmaFECT 1 Transfection Reagent (horizon). Three days after transfection, cells were co-transfected with siRNA and either pIRESneo2-FLAG-Polη or the empty vector using the Neon transfection system (Thermo Fisher Scientific). For Figure 1*C*, XP2SASV3 cells were transfected with pIRESneo2-FLAG-polη, pIRESneo2-FLAG-PolηΔC, or the empty vector using the Neon transfection system (Thermo Fisher Scientific). Twenty-four hours after plasmid transfection, cells were irradiated with 15 J/m^2^ UV-C and incubated for an additional 3 h. Cells were then harvested and resuspended in lysis buffer [20 mM Tris-HCl (pH 7.6), 150 mM KCl, 25% glycerol, 0.5% NP-40, 1.5 mM MgCl_2_, 1× cOmplete Protease Inhibitor Cocktail (Roche), and 1× PhosSTOP phosphatase inhibitor (Roche)]. Soluble materials were separated by centrifugation (17,600*g* for 15 min at 4 °C), and the precipitants were resuspended in micrococcal nuclease buffer [20 mM Tris-HCl (pH 7.6), 100 mM KCl, 0.3 M sucrose, 0.1% Triton X-100, 2 mM MgCl_2_, 1 mM CaCl_2_, 1× EDTA-free cOmplete protease inhibitor cocktail (Roche)] and incubated for 10 min at room temperature with 2.5 U of micrococcal nuclease (Roche; Worthington Biochemical Corporation). The Chromatin-bound materials were collected by centrifugation at 300*g* for 5 min at 4 °C. The soluble and chromatin fractions were mixed with ANTI-FLAG M2 Affinity gel (SIGMA Aldrich) in buffer C [20 mM Tris-HCl (pH 8.0), 100 mM KCl, 10% glycerol, 0.1% Tween 20, 5 mM MgCl_2_, 0.2 mM EDTA, and 0.2 mM phenylmethylsulfonyl fluoride]. After incubation at 4 °C for 2 h, the beads were washed with buffer C, and bound proteins were eluted with 0.4 mg/ml FLAG peptide. SDS-PAGE resolved input and bound fractions and proteins were detected by immunoblotting. Mouse monoclonal cl.5H10 antibody against human Polη was used to detect Polη.

### Anti-FLAG immunoprecipitation with purified proteins

One pmol each of Polη, RAD18, and DNA, where indicated, were mixed with ANTI-FLAG M2 Affinity gel in buffer B containing 20 mM Tris-HCl (pH 8.0), 100 mM NaCl, 10% glycerol, 0.1% NP-40, and 1× EDTA-free cOmplete Protease Inhibitor Cocktail (Roche). After incubation at 4 °C for 3 h, the beads were washed with buffer B, and bound proteins were eluted with 0.4 mg/ml FLAG peptide. Input, bound, and unbound fractions were resolved by SDS-PAGE and proteins were detected by immunoblotting. Anti-Polη133 antibody was used to detect Polη.

### DNA used in anti-FLAG immunoprecipitation

The 5′ protruding DNA was prepared by annealing 14-2 and ND30-2 at a 1:1 moler ratio, and the 3′ protruding DNA was prepared by annealing 16-2 and ND30-2 at a 1:1 moler ratio (Fig. 4*A* and *C*).

### Ni-NTA pull-down assay

One pmol each of Polη and RAD18 were incubated with Ni-NTA magnetic beads (QIAGEN) in 180 μl buffer D (20 mM sodium phosphate (pH 7.2), 150 mM NaCl, 0.1% Triton X-100, 20 mM imidazole and 1× EDTA-free cOmplete Protease Inhibitor Cocktail). After incubation at 4 °C for 2 h, the beads were washed with buffer D, and bound proteins were eluted with 0.4 M imidazole. Input, bound, and unbound fractions were resolved by SDS-PAGE and proteins were detected by immunoblotting.

### Dephosphorylation

FLAG-RAD18 purified from Sf9 cells (290 ng) was mock-treated or treated with 400 units of λPP (New England BioLabs) for 15 min at 30 °C in a 96 μl reaction (1xNEBuffer for PMP and 1 mM MnCl_2_), and used in the Ni-NTA pull-down assay (Fig. 2*D*).

### Anti-Polη immunoprecipitation

Anti-Polη immunoprecipitation was performed as described previously (48). Briefly, Polη was mixed with protein G Sepharose conjugated with a mouse monoclonal anti-Polη antibody, cl.5C6, and then incubated with RAD18. The bound proteins were eluted with 1% SDS.

### Electrophoretic mobility shift assay

EMSAs were performed as described previously (13). Briefly, the reaction mixture (10 μl) containing 25 mM potassium phosphate buffer (pH 7.4), 0.2 mg/ml BSA, 5 mM dithiothreitol, 2.5% glycerol, 15 mM KCl, 20 mM NaCl, 5′-^32^P-labeled DNA (5 fmol, labeled primer DNA was mixed with unlabeled template DNA at a 1:1.2 molar ratio), poly dI-dC (1 ng), and the indicated amount of ^His^PolηΔC or PolηΔC was incubated on ice for 15 min. The mixtures were directly loaded onto 4% non-denaturing polyacrylamide gels (acrylamide:bis-acrylamide, 79 : 1) and electrophoresed at 8 V/cm at 4 °C in TAE buffer (6 mM Tris-HCl (pH 7.5), 5 mM sodium acetate, and 0.1 mM EDTA). The percentage of the probe DNA bound to PolηΔC was determined using Typhoon 9000 (GE).

### UV sensitivities

Cells in 6-well plates were irradiated with the indicated dose of UVC, incubated with 1 mM caffeine for 4 days, and viabilities were measured using CellTiter 96 AQ_ueous_ One Solution Cell Proliferation Assay (MTS) (Promega).

### DNA-polymerase assay

The 5′-^32^P-labeled primer-template DNA was prepared by mixing DNA oligonucleotide 16-1 labeled at its 5′ end with ND30, CPD30, or (6-4)30 at a 1:1.2 moler ratio. Singly-primed M13 mp18 ssDNA was prepared by mixing the 36-mer M13 primer (complementary to nucleotides 6330-6295) labeled at its 5′ end with the M13 mp18 ssDNA at a 1:1.25 moler ratio (Fig. S7*D*). DNA-polymerase assays were performed as described previously (8). Briefly, the indicated amount of Polη was incubated with 32 nM (Fig. S7*D* and *E*) or 40 nM (Figs. S2*D* and S3*C*) of the 5′-^32^P-labeled primer-template DNA in a buffer (10 μl) containing 40 mM Tris-HCl (pH 8.0), 5 mM MgCl_2_, 100 μM each of the four dNTPs, 10 mM DTT, 250 mg/ml BSA, 60 mM KCl, and 2.5% glycerol. Reactions were performed at 37 °C for 15 min and terminated by adding 10 μl of formamide followed by boiling. Products were electrophoresed on a denaturing gel and visualized by Typhoon 9000 (GE).

### AlphaFold 3 prediction

To model the DNA–PolηΔC complex, the protein sequence of PolηΔC and two DNA sequences (5′-ACTGACTGTATGCTGAAGA-3′ and 5′-CTCGTCAGCATCTTCAGCATACAGTCAGTG-3′) were submitted to the AlphaFold Server (56). To model the RAD18–PolηΔC complex, the protein sequences of PolηΔC and RAD18 were submitted to the AlphaFold Server. Molecular graphics and analyses were performed using UCSF ChimeraX (57).

### Statistical analysis

GraphPad Prism 9 software (GraphPad Software, https://www.graphpad.com/) was used for statistical analysis. Error bars show the SD of the data from at least three independent experiments. Linear regression analyses were performed using Prism 9 software, and LD50 values were calculated.

## Data availability

The data in this article will be shared upon reasonable request to the corresponding author.

## Supporting information

This article contains supporting information.

## Conflict of interest

The authors declare that they have no conflicts of interest with the contents of this article.
