# Supplementary material for: Overlapping RAD18-and DNA-binding interfaces in DNA polymerase η contribute to UV-induced DNA damage tolerance
Source: J Biol Chem. 2026 Sep 15;302(11):113555. doi: 10.1016/j.jbc.2026.113555 (PMC13678009; doi:10.1016/j.jbc.2026.113555)
Supplement: Supplementary file 1 [file mmc1.pdf]

SUPPORTING INFORMATION

Title

Overlapping RAD18- and DNA-binding interfaces in DNA polymerase η contribute to UV-induced DNA damage tolerance

Authors

Rika Kusumoto-Matsuo[‡], Yuji Masuda[‡], Rie Kanao[‡], Shigenori Iwai and Chikahide Masutani

Figure S1-S11

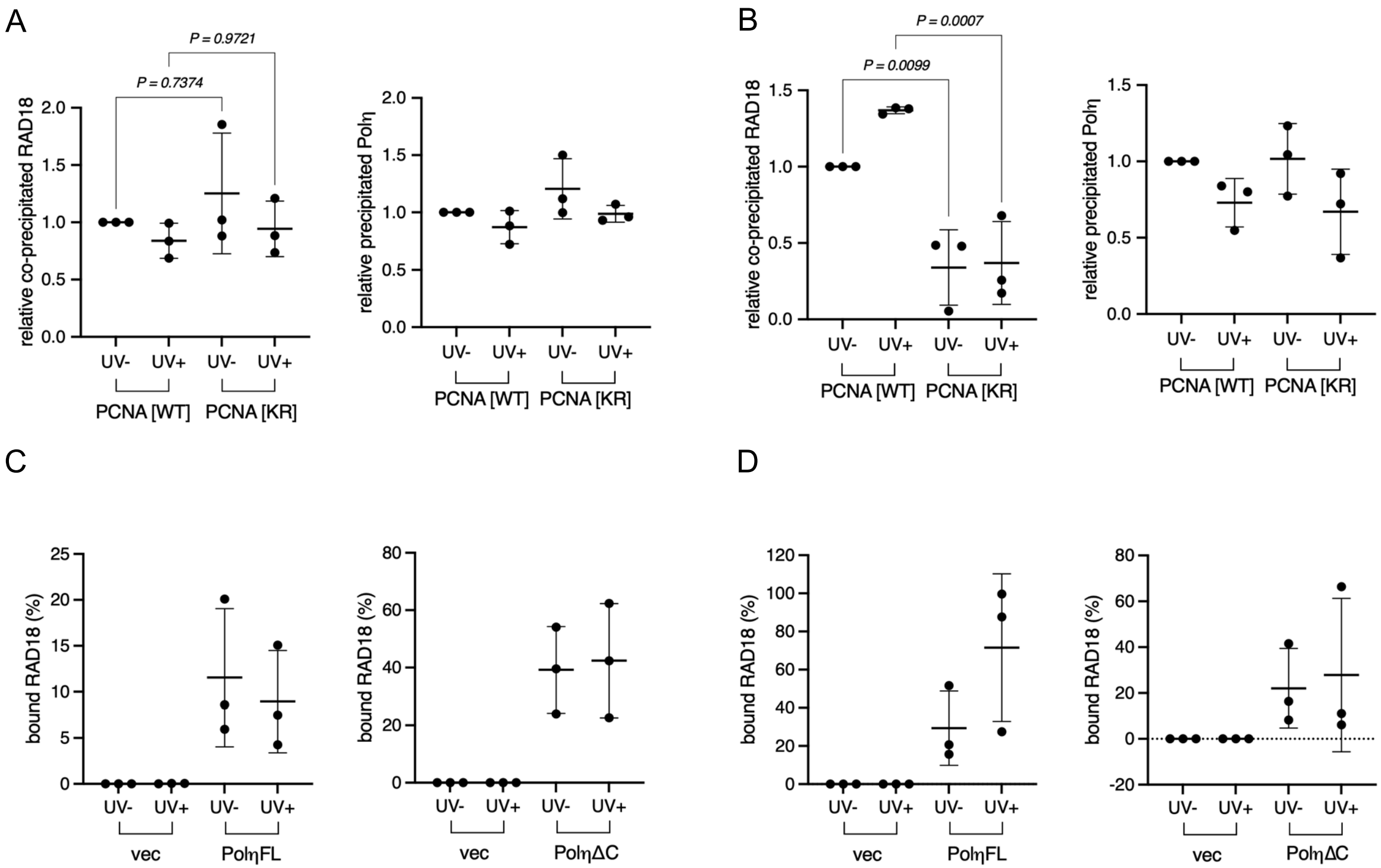

Figure S1. Quantification of the immunoprecipitation data presented in Figure 1. (A-D) Quantification of the immunoprecipitation shown in Fig. 1A-D, respectively. (A and B) RAD18 and Polη in the immunoprecipitated complexes were quantified and expressed as a percentage of input. Values were further normalized to those obtained in assays expressing PCNA[WT] in unirradiated cells. (C and D) RAD18 in the immunoprecipitated complexes was quantified and expressed as a percentage of input. Three independent experiments were performed. For panels A and B, individual values are shown together with the mean ± SD. Statistical analysis was performed using ordinary one-way ANOVA (panel A, $F_{3,8}$ = 1.030, P = 0.4294; panel B, $F_{3,8}$ = 22.41, P = 0.0003) followed by Tukey's multiple comparison test, and p values are indicated. For panels C and D, individual values from independent experiments are presented to illustrate the reproducibility of the observed interactions.

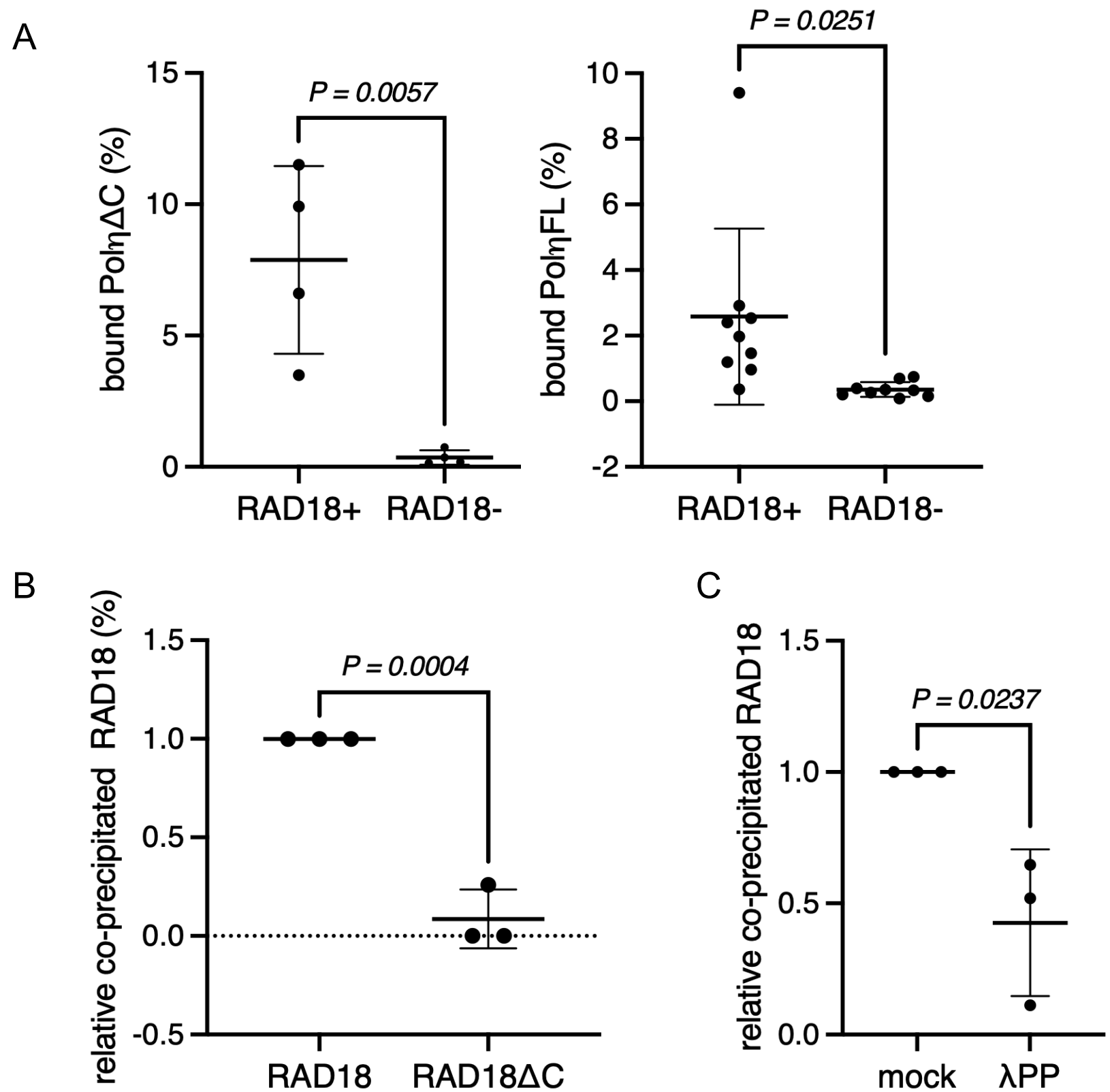

Figure S2. Quantification of the immunoprecipitation data presented in Figure 2.
(A-C) Quantification of the immunoprecipitation and Ni-NTA pulldown shown in Fig. 2B–D, respectively. (A) PolηFL and PolηΔC in the immunoprecipitated complexes in Fig. 2B were quantified and expressed as a percentage to input. (B) RAD18 and RAD18ΔC in the bead-bound complexes in Fig. 2C were quantified and expressed as a percentage to input. Values were further expressed relative to those in assays with RAD18. (C) RAD18 in the bead-bound complexes in Fig. 2D were quantified and expressed as a percentage to input. The ratio to that in assays with RAD18 in mock treated cells was expressed. (A-C) The experiments were repeated three times at least, and individual values were plotted. Data are presented as the mean ± SD. Statistical analysis was performed using an unpaired two-tailed *t* test. *P* values are indicated.

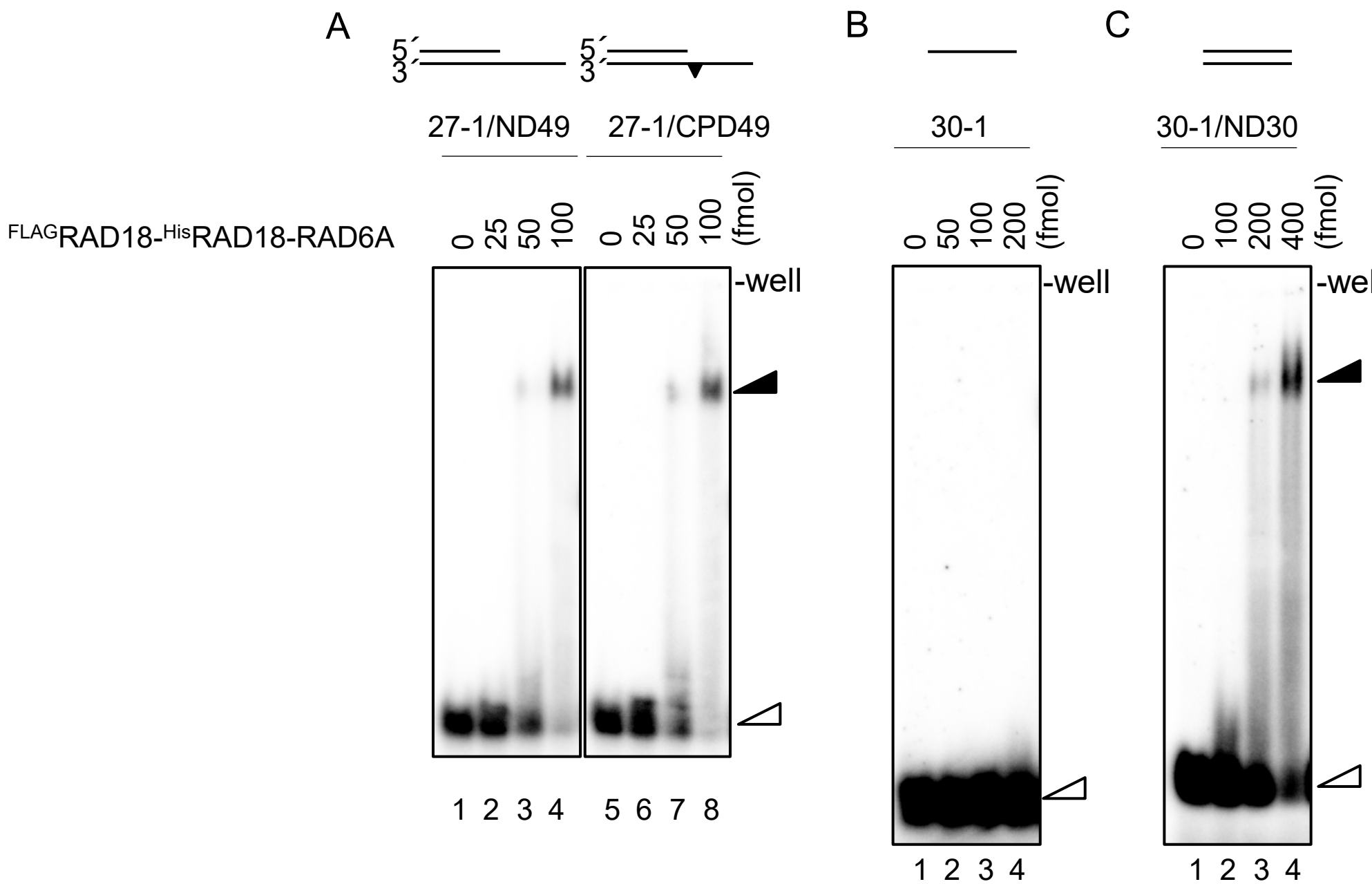

Figure S3. Binding activity of RAD18 complex to primer/template DNA, ssDNA and dsDNA.
EMSA. Increasing amount of FLAGRAD18-HisRAD18-RAD6A were incubated with 5 fmol of the ^{32}P-labelled 27-1/ND49 or 27-1/CPD49 (A), 30-1 (B) and 30-1/ND30 (C). The resulting DNA-protein complexes were separated by PAGE. White arrowhead: free DNA, black arrowhead: DNA-protein complex.

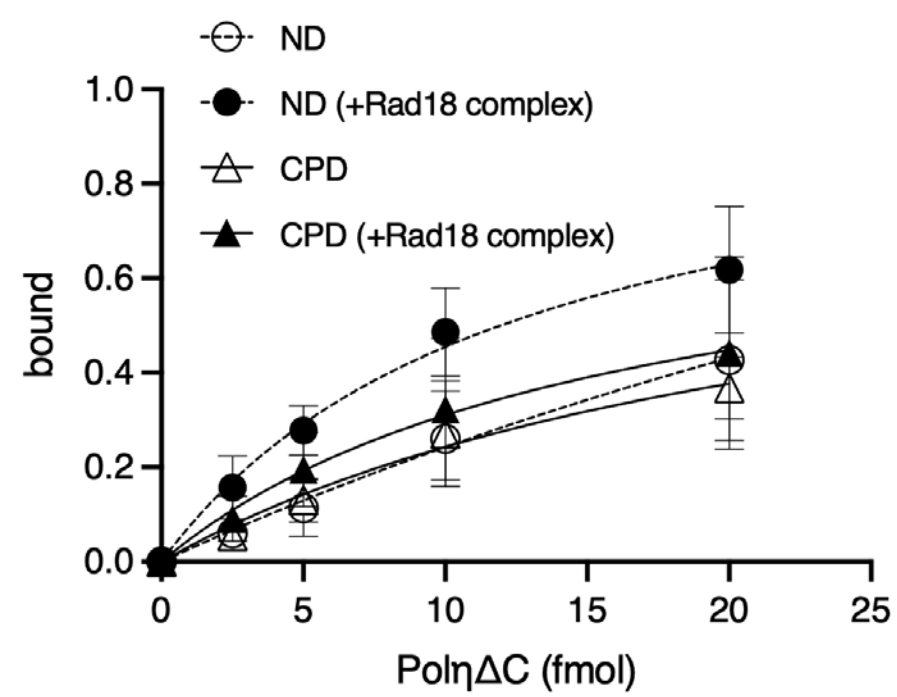

P values	2.5 fmol	5 fmol	10 fmol	20 fmol
ND vs ND (+Rad18)	0.1087	0.0268	0.0851	0.4055
CPD vs CPD (+Rad18)	0.2080	0.2079	0.6049	0.5110

Figure S4. Quantification of polηΔC binding to DNA by EMSA. The amount of the labelled probe complexed with polηΔC was calculated as a fraction of the total DNA for each lane in Figure 3A. Open circles: lanes 1–5; closed circles: lanes 6–10; open triangles: lanes 11–15; closed triangles: lanes 16–20. The mean values and standard errors were calculated from five independent experiments. Statistical analysis was performed using an unpaired two-tailed *t* test. *P* values are shown.

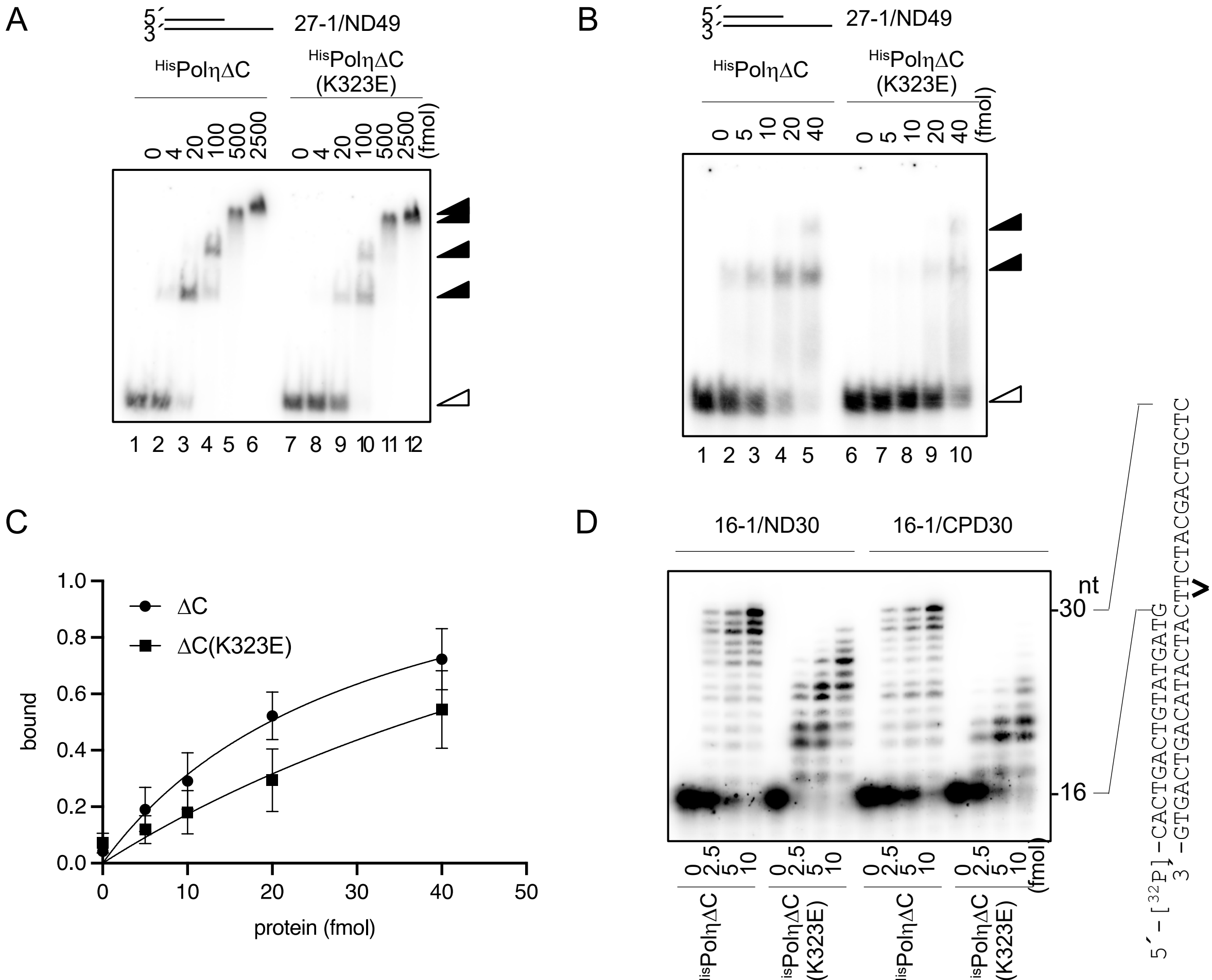

Figure S5. Analysis of PolηΔC and PolηΔC(K323E).

(A-C) EMSA. (A-B) Increasing amount of HisPolηΔC or HisPolηΔC(K323E) was incubated with 5 fmol of the ^{32}P-labelled 27-1/ND49. The resulting DNA-protein complexes were separated by PAGE. White arrowhead: free DNA, black arrowhead: DNA-protein complex. (C) The amount of the labeled substrate complexed with PolηΔC or PolΔC(K323E) was calculated as a fraction of the total DNA for each lane in (B). The means ± SDs were calculated from eight independent experiments. Non-linear regression with one site-specific binding is used to draw the curve using GraphPad Prism9. (D) DNA polymerase assay. HisPolηΔC or HisPolηΔC(K323E) was incubated with the ^{32}P-labelled 16-1/CPD30 or 16-1/ND30. The DNA products were separated by Urea-PAGE.

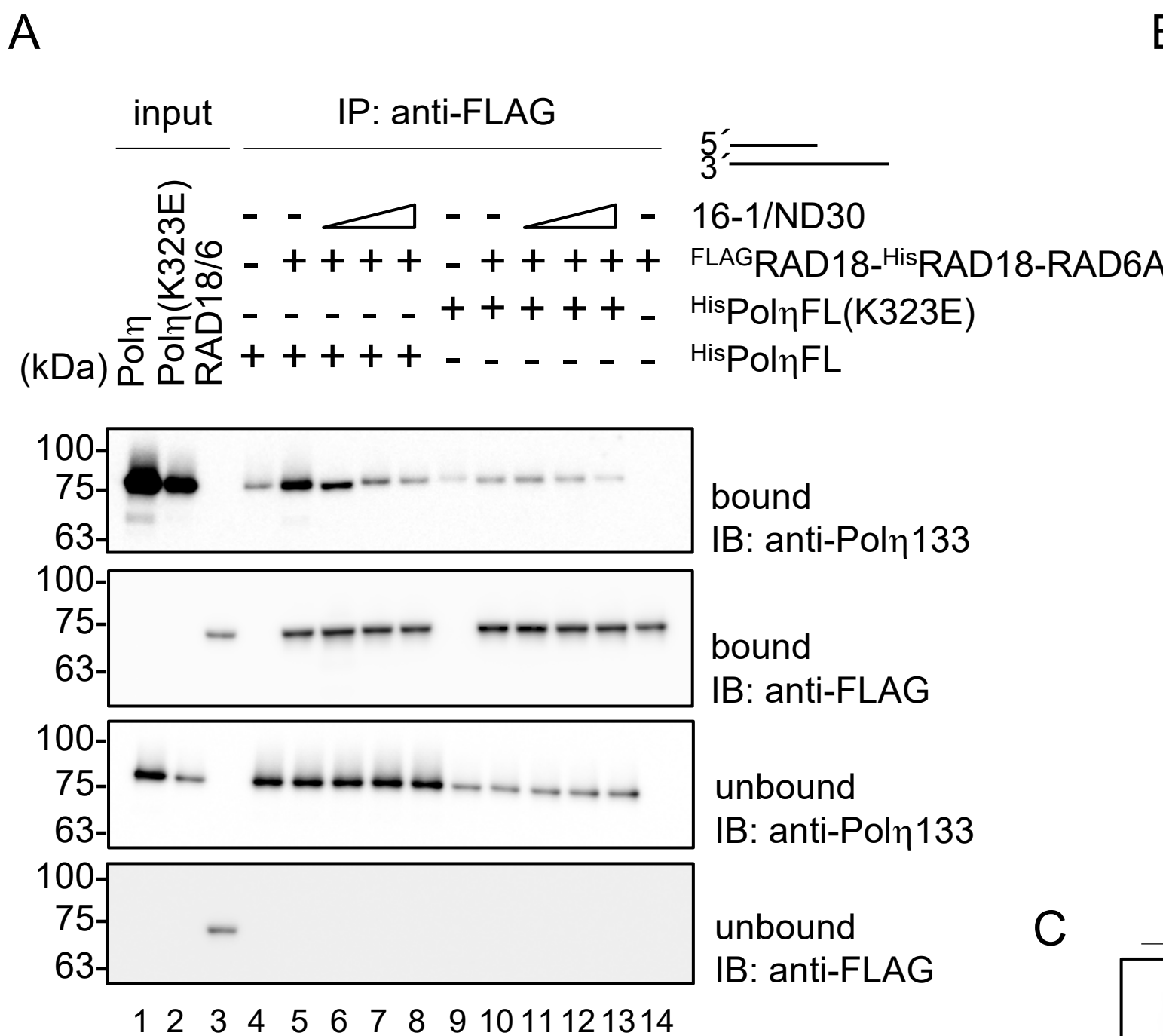

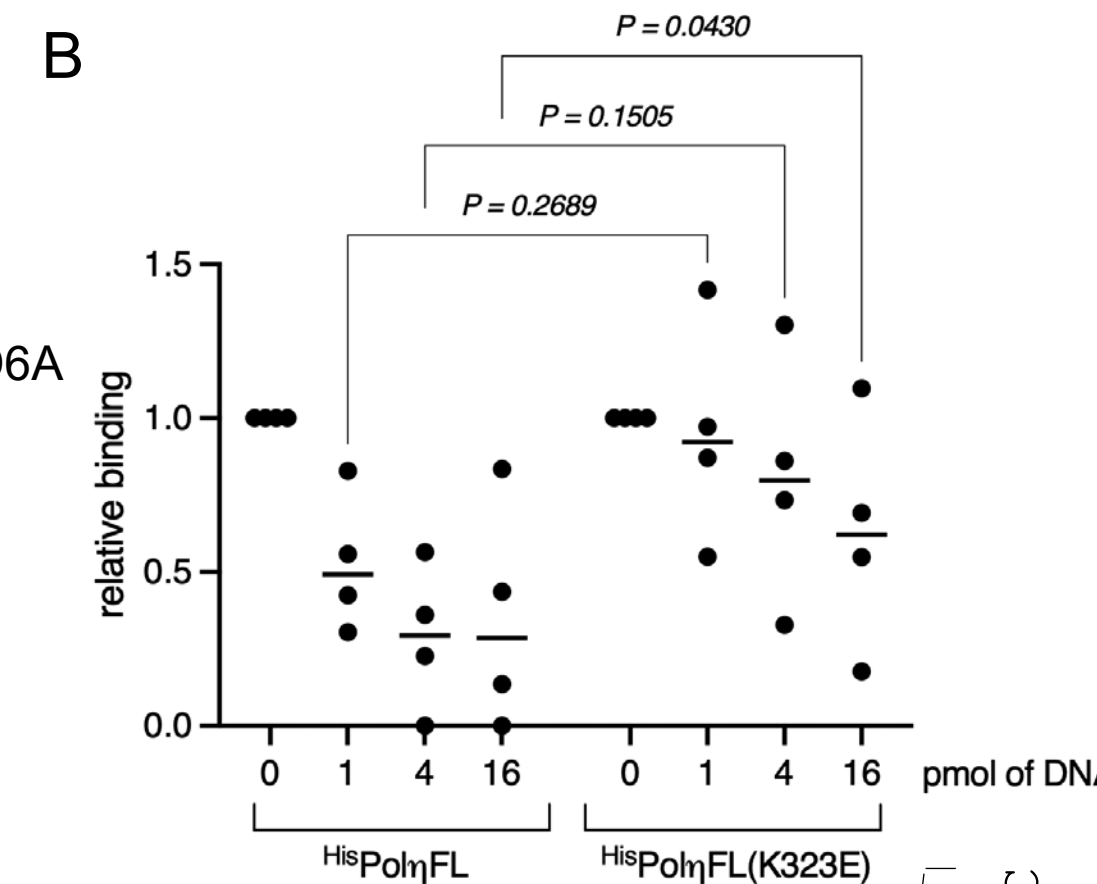

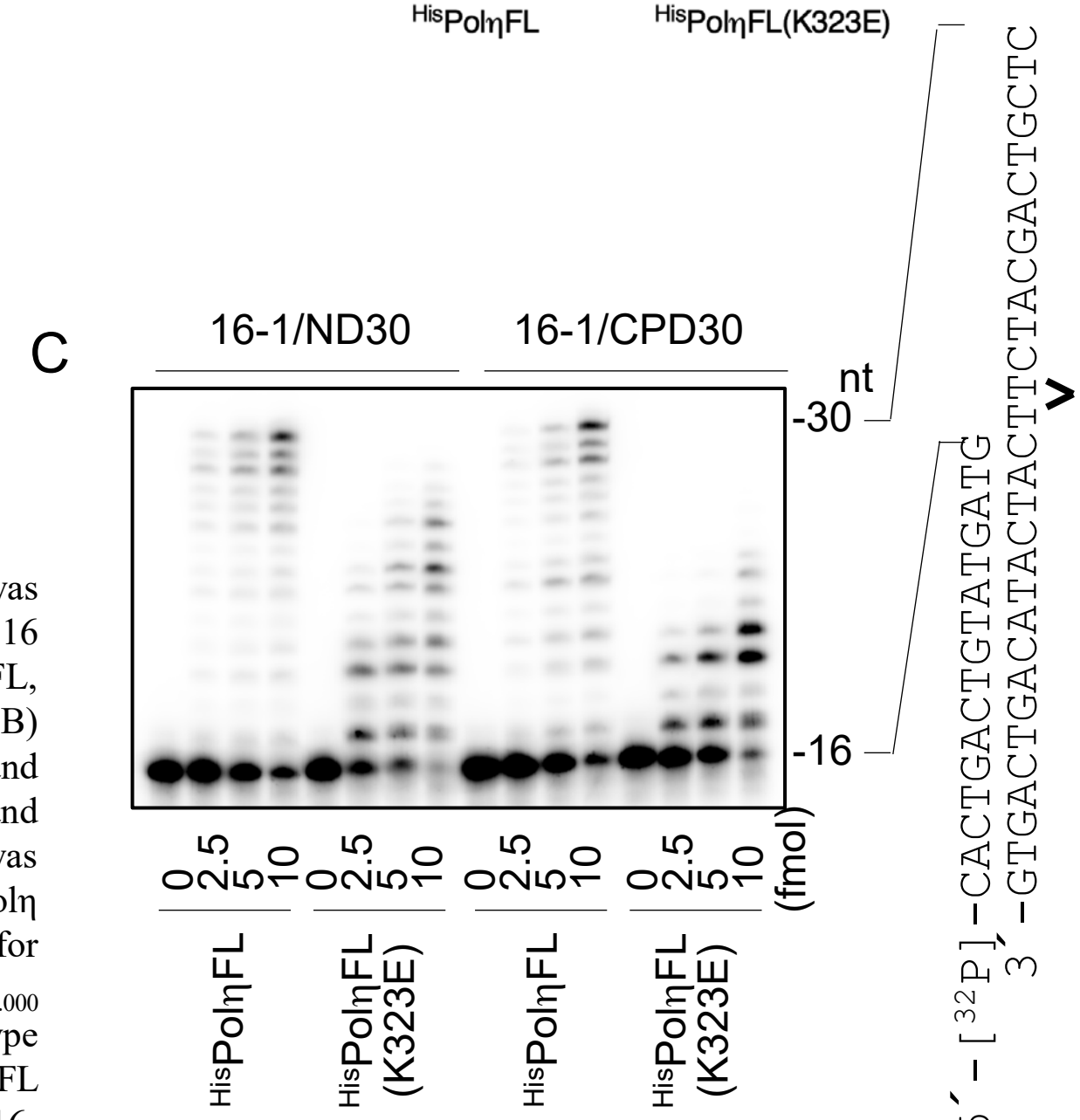

Figure S6. Analysis of PolηFL(K323E).

(A) Anti-FLAG immunoprecipitation. FLAGRAD18-HisRAD18-RAD6A was incubated with either HisPolηFL or HisPolηFL(K323E) in the presence of 1, 4 or 16 pmol of 5′-protruding DNA (16-1/ND30). Lanes 1, 2 and 3: 10% of PolηFL, PolηFL(K323E) and 20% of RAD18 input to the bound fraction, respectively. (B) The PolηFL or PolηFL(K323E) in the immune complex were quantified and expressed as a ratio to it without DNA. Experiment was repeated four times and individual values were plotted with average bars. Statistical analysis was performed using RM two-way ANOVA, with DNA concentration and Polη genotype as factors and both factors matched. Significant effects were observed for DNA concentration ($F_{1.629, 4.888} = 6.567$, $P = 0.0442$) and Polη genotype ($F_{1.000, 3.000} = 14.71$, $P = 0.0312$), but not for the DNA concentration × Polη genotype interaction ($F_{1.120, 3.359} = 6.304$, $P = 0.0772$). (C) DNA polymerase assay. HisPolηFL or HisPolηFL(K323E) was incubated with the 32P-labelled 16-1/CPD30 or 16-1/ND30. The DNA products were separated by Urea-PAGE.

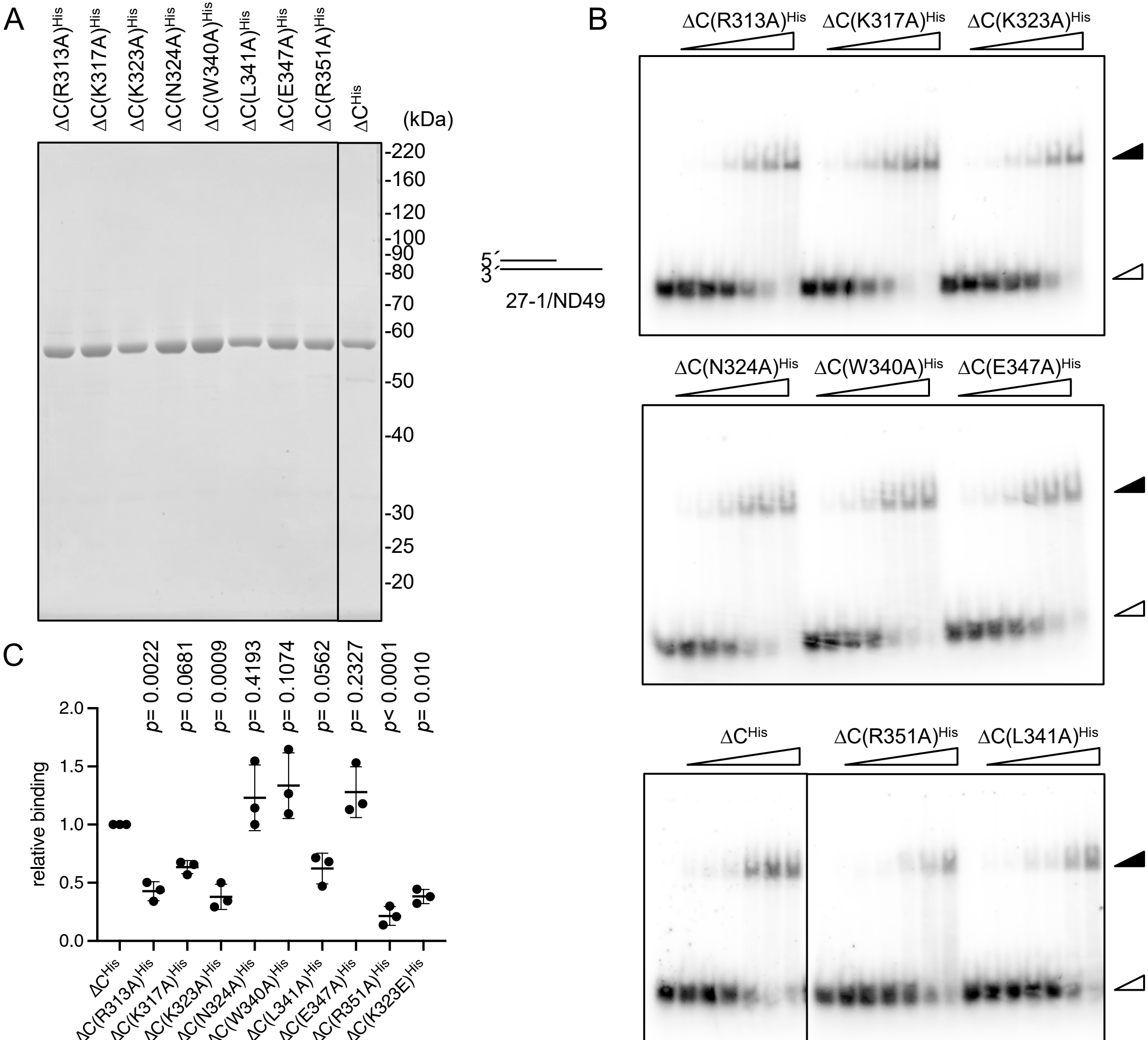
A
ΔC(R313A)His
ΔC(K317A)His
ΔC(K323A)His
ΔC(N324A)His
ΔC(W340A)His
ΔC(L341A)His
ΔC(E347A)His
ΔC(R351A)His
ΔCHis
(kDa)
-220
-160
-120
-100
-90
-80
-70
-60
-50
-40
-30
-25
-20
B
5´
3´
27-1/ND49
ΔC(R313A)His
ΔC(K317A)His
ΔC(K323A)His
ΔC(N324A)His
ΔC(W340A)His
ΔC(E347A)His
ΔCHis
ΔC(R351A)His
ΔC(L341A)His
C
p= 0.0022
p= 0.0681
p= 0.0009
p= 0.4193
p= 0.1074
p= 0.0562
p= 0.2327
p< 0.0001
p= 0.010
relative binding
2.0
1.5
1.0
0.5
0.0
ΔCHis
ΔC(R313A)His
ΔC(K317A)His
ΔC(K323A)His
ΔC(N324A)His
ΔC(W340A)His
ΔC(L341A)His
ΔC(E347A)His
ΔC(R351A)His
ΔC(K323E)His

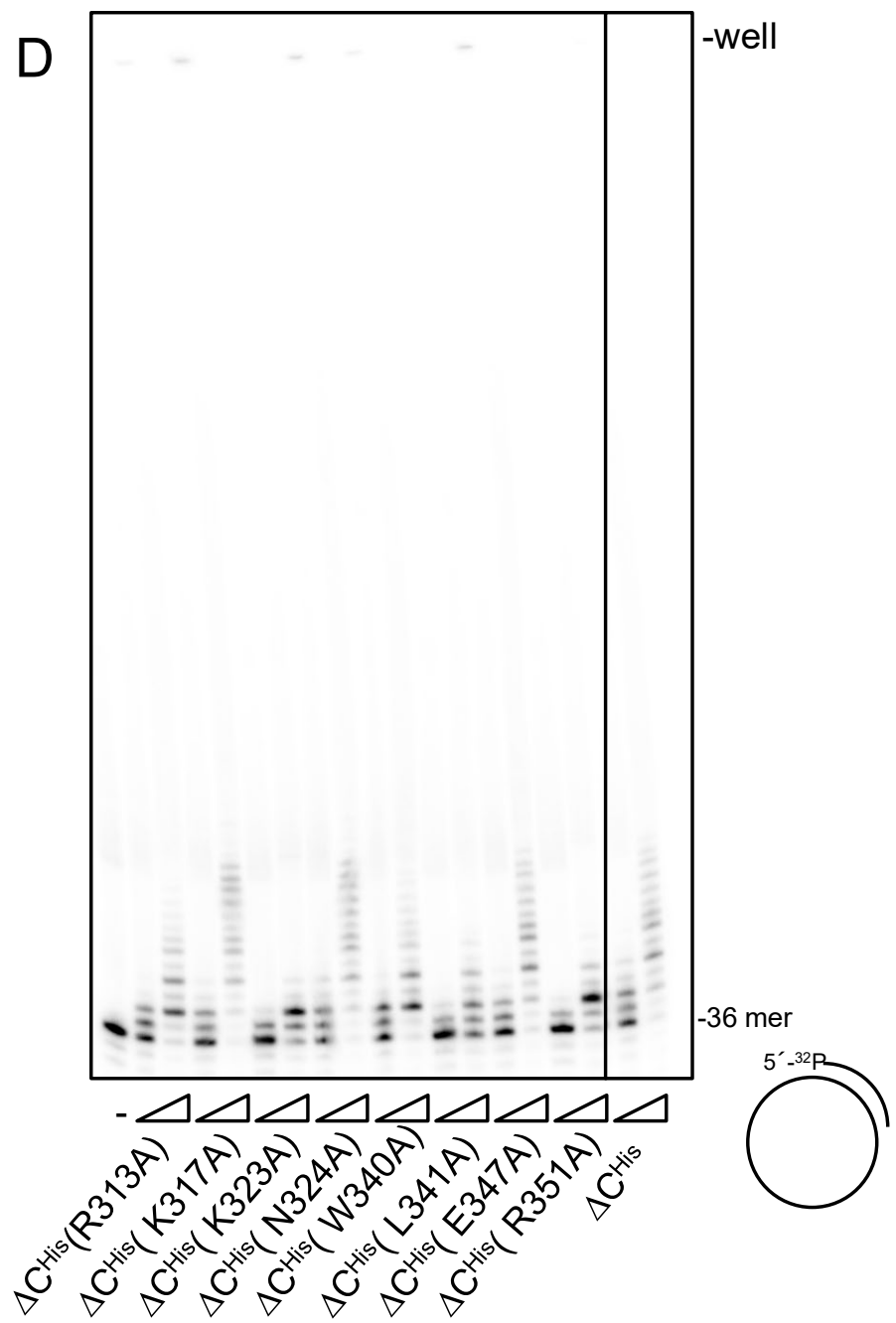

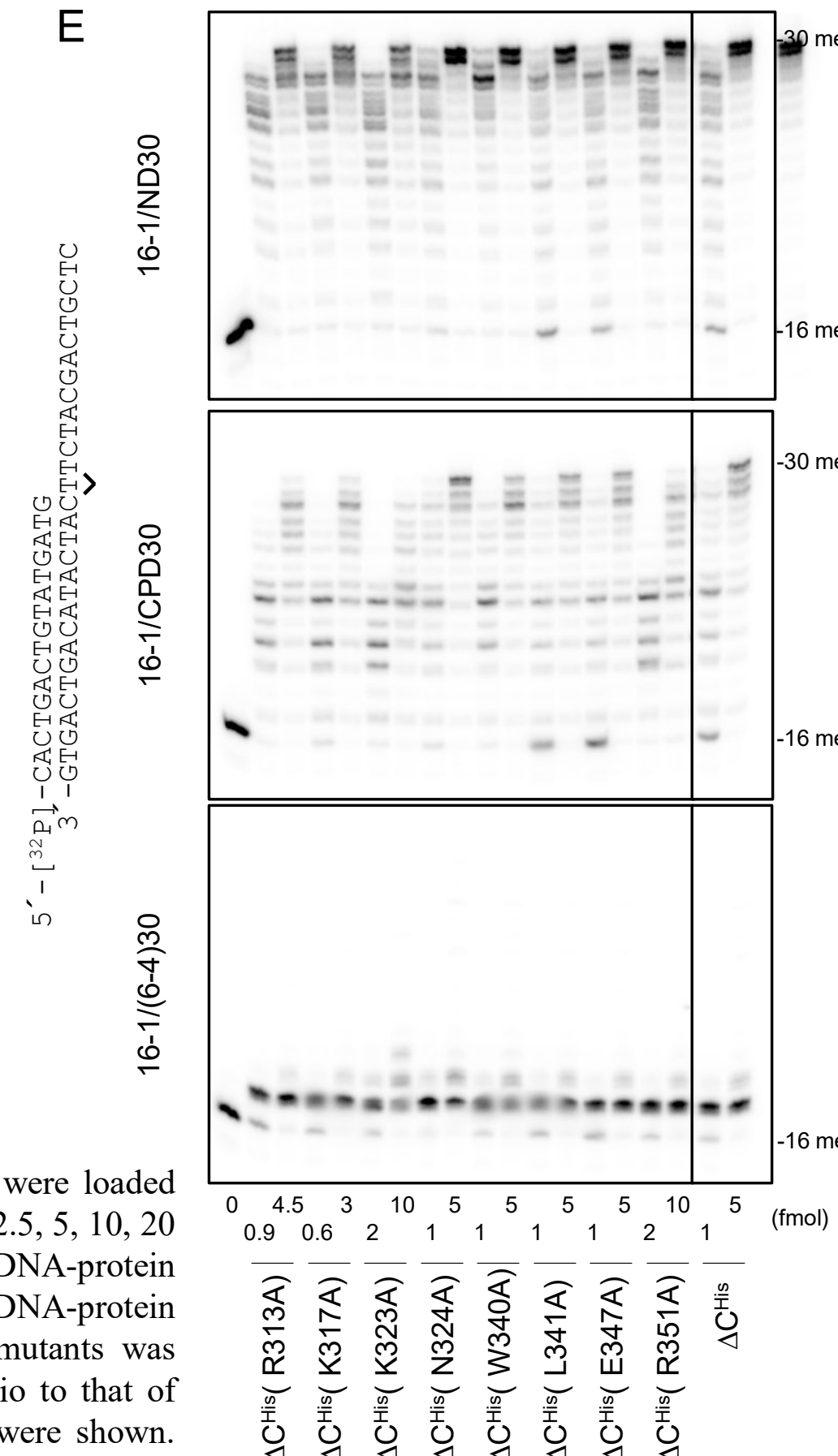

Figure S7. DNA binding and DNA polymerase activities of PolηΔC mutants.

(A) Purified recombinant PolηΔC. Five hundred nanograms of each indicated protein were loaded onto an SDS-PAGE, and the gel was stained with CBB. (B) EMSA. PolηΔC^{His} (0, 1.25, 2.5, 5, 10, 20 or 40 fmol) was incubated with 5 fmol of the ^{32}P-labelled 27-1/ND49. The resulting DNA-protein complexes were separated by PAGE. White arrowhead: free DNA, black arrowhead: DNA-protein complex. (C) The amount of the labeled substrate complexed with PolηΔC or its mutants was calculated as a fraction of the total DNA for each lane in (B), and expressed as a ratio to that of PolηΔC. Individual values of 5, 10 and 20 fmol were plotted and the means ± SDs were shown. Statistical analysis was conducted by one-way ANOVA with Dunnett's multiple comparisons test, comparing each group to control (F = 20.89, P < 0.0001). p values were shown. (D and E) DNA polymerase assay. (D) PolηΔC^{His} or its mutants (0, 5 or 25 fmol) was incubated with 320 fmol of [^{32}P] singly primed M13 mp18 ssDNA. (E) [^{32}P]16-1/ND30 (upper), [^{32}P]16-1/CPD30 (middle) and [^{32}P]16-1/(6-4)30 (lower) were used as substrates. PolηΔC^{His} or its mutants (as indicated in the figure) was incubated with 320 fmol of the substrate.

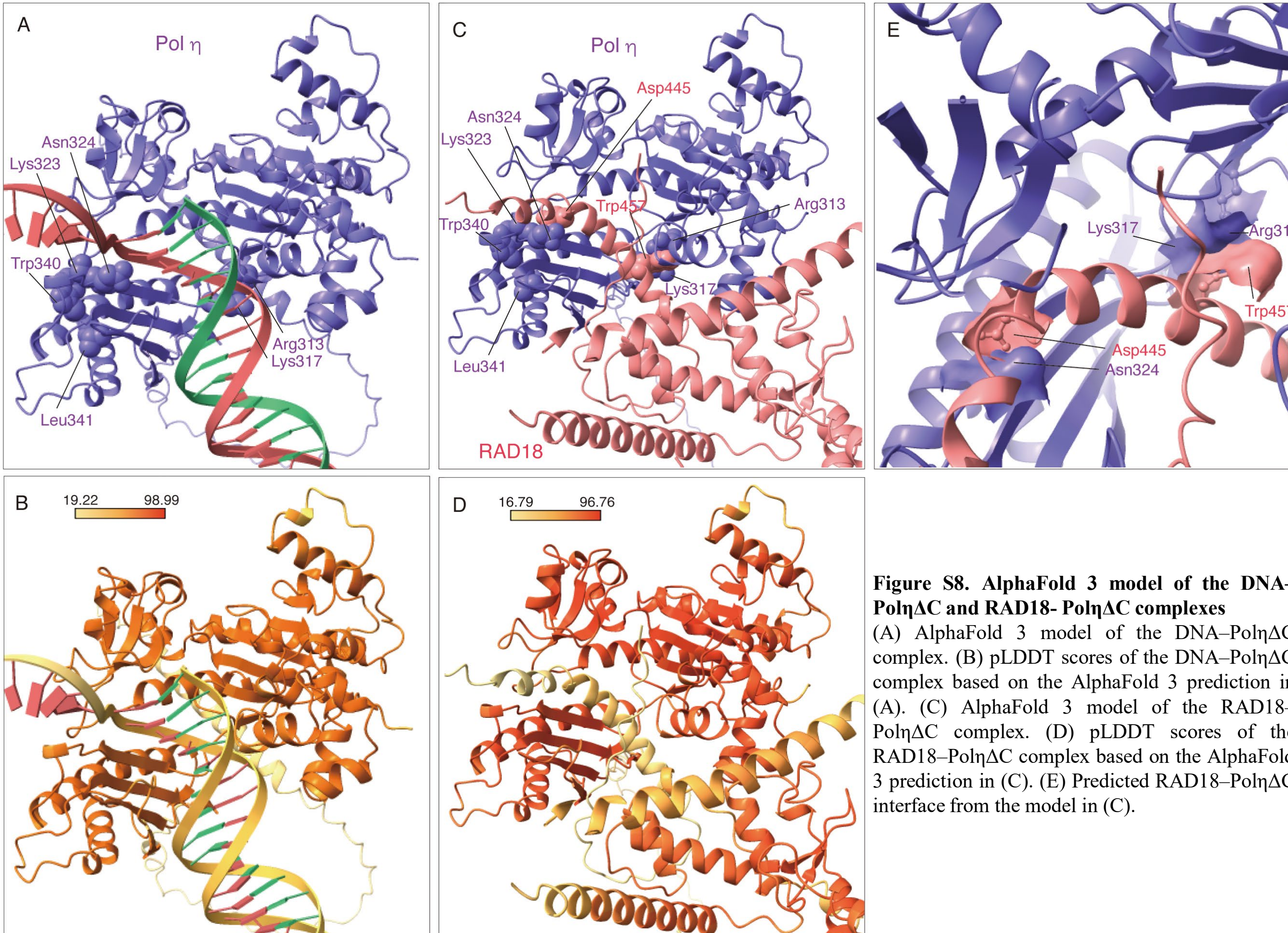

Figure S8. AlphaFold 3 model of the DNA–PolηΔC and RAD18- PolηΔC complexes
(A) AlphaFold 3 model of the DNA–PolηΔC complex. (B) pLDDT scores of the DNA–PolηΔC complex based on the AlphaFold 3 prediction in (A). (C) AlphaFold 3 model of the RAD18–PolηΔC complex. (D) pLDDT scores of the RAD18–PolηΔC complex based on the AlphaFold 3 prediction in (C). (E) Predicted RAD18–PolηΔC interface from the model in (C).

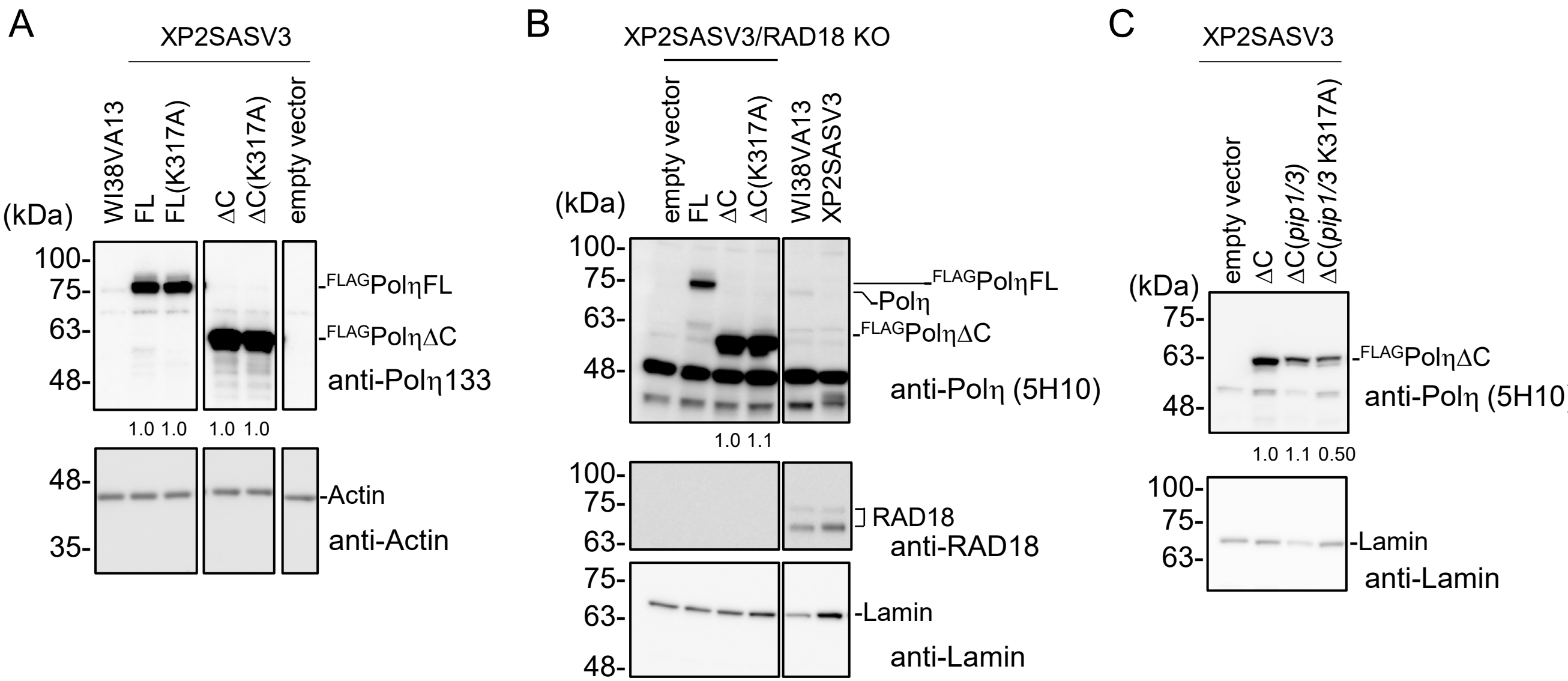

Figure S9. Protein expression of Polη(K317A)

(A, B and C) Western blot analysis of whole cell lysate. (A) XP-V cells stably expressing RAD18 binding mutants of PolηFL(K317A) or PolηΔC(K317A). (B) XP2SASV3/RAD18 KO cells stably expressing PolηFL, PolηΔC or PolηΔC(K317A). (C) XP-V cells stably expressing RAD18 binding and PCNA binding mutants of PolηΔC (K317A with F443A/L444A/F483A/F484A). (A-C) POLη signals were normalized to Actin (A) or Lamin (B and C) and expressed relative to the sample without point mutation, which was assigned a value of 1.0.

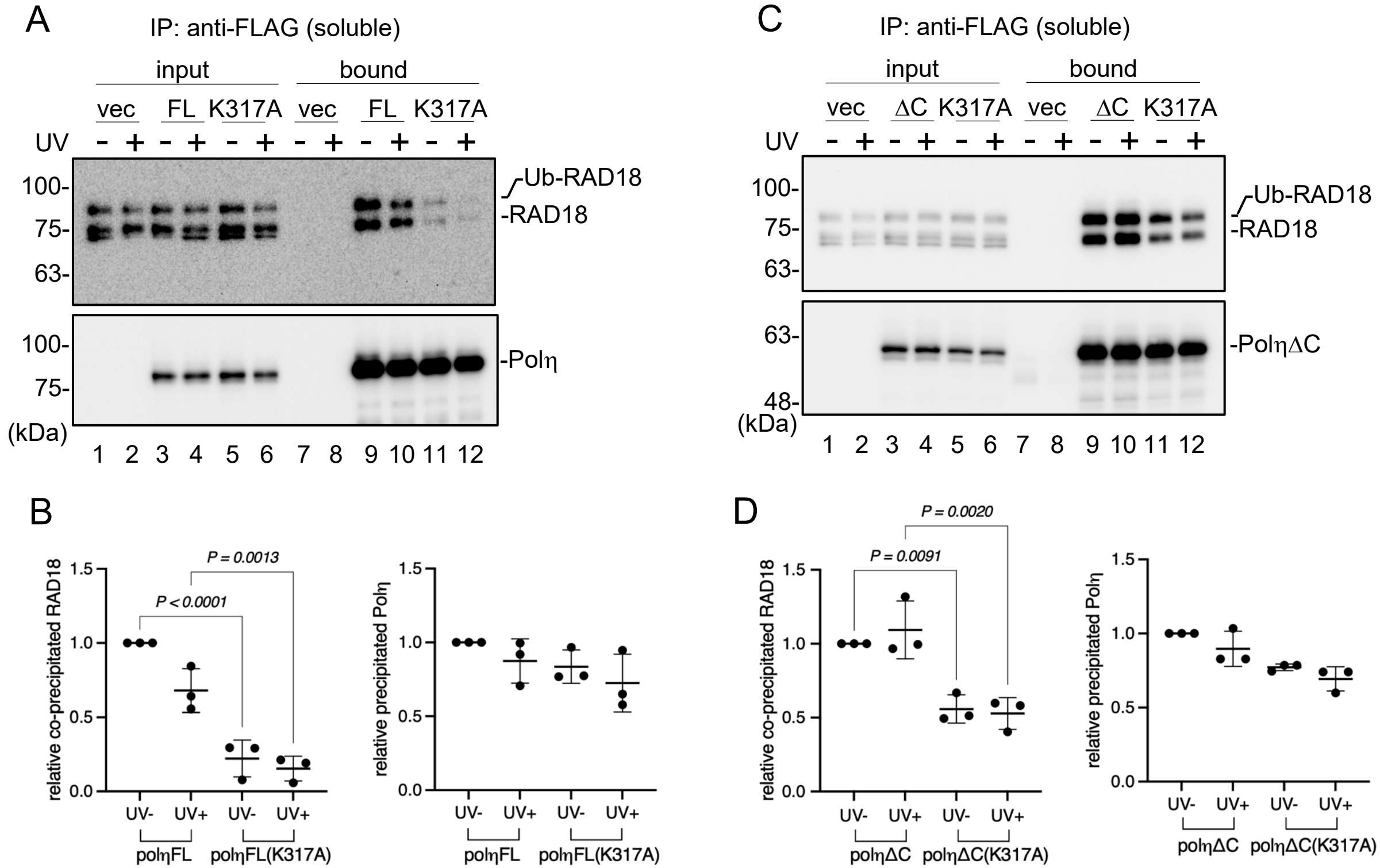

Figure S10. Cellular functions of K317 of Polη

Anti-FLAG immunoprecipitation. (A and B) XP2SASV3 cells were transfected with either an empty vector or a vector to express ^{FLAG}PolηFL or ^{FLAG}PolηFL(K317A). Cells were irradiated with 15 J/m^2 UV-C or mock-treated and incubated for 3 h. ^{FLAG}PolηFL or ^{FLAG}PolηFL(K317A) in detergent-soluble were immunoprecipitated with anti-FLAG antibody. Immunoprecipitated complexes (lanes 7-12) and 5% of input (lanes 1-6) used in immunoprecipitation were analyzed by Western blotting with indicated antibodies (A). RAD18 and ^{FLAG}PolηFL or ^{FLAG}PolηFL(K317A) in the immunoprecipitated complexes were quantified. Values were further normalized to those in assays expressing ^{FLAG}PolηFL in unirradiated cells. The experiment was repeated three times, and individual values were plotted along with the mean ± SD. (B left) Statistical analysis was conducted ($F_{3,8}$ = 43.32, $P < 0.0001$, one-way ANOVA with Tukey's multiple comparison test). p values are shown. (C and D) XP2SASV3 cells were transfected with either an empty vector or a vector to express ^{FLAG}PolηΔC or ^{FLAG}PolηΔC (K317A) and treated as indicated in A and B. (D left) Statistical analysis was conducted ($F_{3,8}$ = 17.58, $P = 0.0007$, one-way ANOVA with Tukey's multiple comparison test). p values are shown.

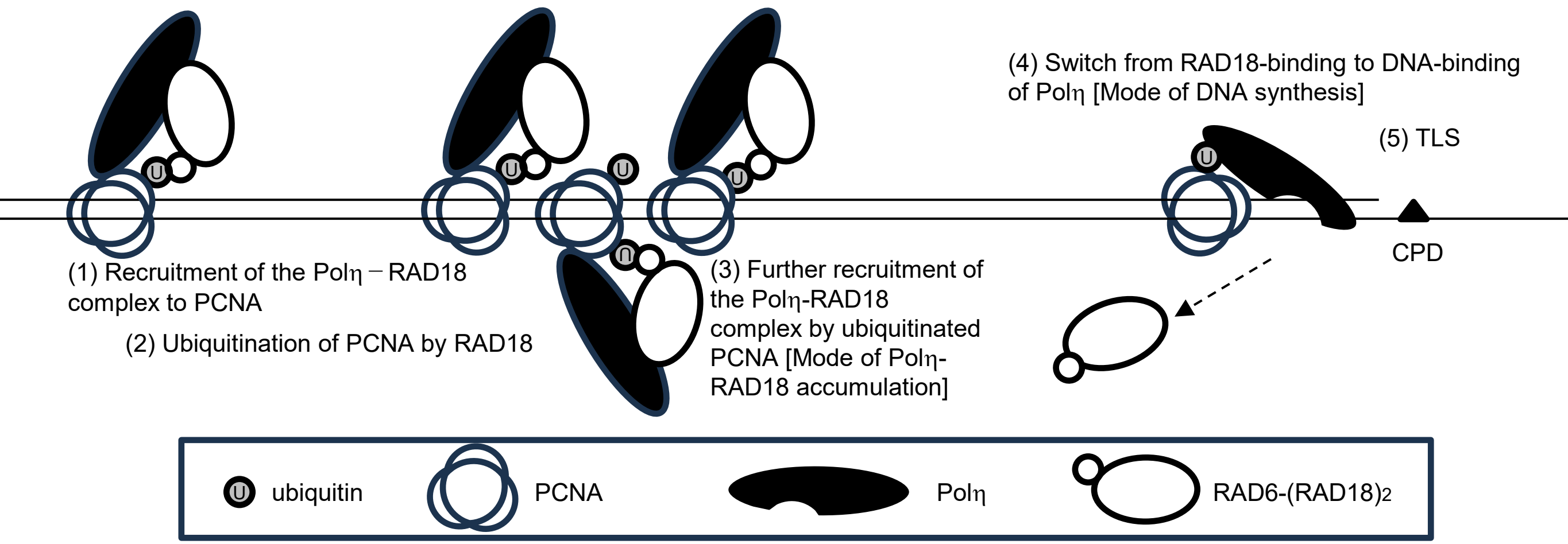

Figure S11. A model for Polη recruitment to the primer-template
When the replicative DNA polymerase encounters an obstacle that blocks further DNA synthesis, the Polη–RAD18 complex is recruited to the stalled replication site (1), where it promotes PCNA mono-ubiquitination (2). Monoubiquitinated PCNA then facilitates further recruitment of the Polη–RAD18 complex (3). Once Polη is loaded onto the stalled primer–template, RAD18 dissociates from Polη (4), allowing Polη to initiate TLS (5).
